# Mapping the Conformational Dynamics and Pathways of Spontaneous Steric Zipper Peptide Oligomerization

**DOI:** 10.1371/journal.pone.0019129

**Published:** 2011-05-03

**Authors:** Dirk Matthes, Vytautas Gapsys, Venita Daebel, Bert L. de Groot

**Affiliations:** 1 Computational Biomolecular Dynamics Group, Max-Planck-Institute for Biophysical Chemistry, Göttingen, Germany; 2 Solid-State NMR, Max-Planck-Institute for Biophysical Chemistry, Göttingen, Germany; National Institute for Medical Research, Medical Research Council, United Kingdom

## Abstract

The process of protein misfolding and self-assembly into various, polymorphic aggregates is associated with a number of important neurodegenerative diseases. Only recently, crystal structures of several short peptides have provided detailed structural insights into 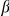-sheet rich aggregates, known as amyloid fibrils. Knowledge about early events of the formation and interconversion of small oligomeric states, an inevitable step in the cascade of peptide self-assembly, however, remains still limited.

We employ molecular dynamics simulations in explicit solvent to study the spontaneous aggregation process of steric zipper peptide segments from the tau protein and insulin in atomistic detail. Starting from separated chains with random conformations, we find a rapid formation of structurally heterogeneous, 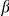-sheet rich oligomers, emerging from multiple bimolecular association steps and diverse assembly pathways. Furthermore, our study provides evidence that aggregate intermediates as small as dimers can be kinetically trapped and thus affect the structural evolution of larger oligomers.

Alternative aggregate structures are found for both peptide sequences in the different independent simulations, some of which feature characteristics of the known steric zipper conformation (e.g., 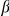-sheet bilayers with a dry interface). The final aggregates interconvert with topologically distinct oligomeric states exclusively via internal rearrangements.

The peptide oligomerization was analyzed through the perspective of a minimal oligomer, i.e., the dimer. Thereby all observed multimeric aggregates can be consistently mapped onto a space of reduced dimensionality. This novel method of conformational mapping reveals heterogeneous association and reorganization dynamics that are governed by the characteristics of peptide sequence and oligomer size.

## Introduction

The assembly of polypeptides and proteins into 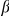-sheet rich aggregates termed amyloid plaques and fibrils is known to be associated with several severe diseases in vivo [1]–[5].

The morphology and molecular structure of mature amyloid fibrils have been investigated extensively in vitro as many polypeptide chains form fibrillar aggregates with cross-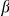 conformation [6]. The application of a wide range of different diffraction and spectroscopy techniques [7]–[14] has helped to elucidate various aspects of fibril architecture and has confirmed molecular polymorphism on several structural levels of filament organization [6], [12], [15]–[17].

However, the mechanistic details and underlying energetics of amyloid fibril formation and the appreciable multitude of conformational states, starting from entities as small as monomers are not well understood [1], [2], [4], [18].

Specifically, the initial and early stages, marked by the population of various soluble oligomeric species still await a consistent and unified description [15], [18]–[21]. Recent advances in the field have contributed to the discovery of a rich variety of amyloid precursors and oligomeric states [22]–[25], yet the structurally heterogeneous and transient nature of these aggregates still does not permit precise experimental characterization.

Analyses of experimentally observed aggregation kinetics suggest different mechanistic explanations for possible pathways and rate-limiting steps of amyloid fibril formation [11], [18]–[20], [26]. But it has not been possible to directly probe the nucleation event in amyloid aggregation so far. While it remains controversial if all observed types of oligomers are indeed on-pathway intermediates [4], [15], [21], an increasing amount of experimental evidence points to soluble protein oligomers as the primary cause of cell impairment and dysfunction in the pathogenesis of neurodegenerative diseases and various amyloidoses [3], [15], [21]–[25], [27].

A mechanistic interpretation of the multi-staged aggregation process and a structural characterization of key intermediates therefore pose an essential challenge, also with regard to the identification and application of therapeutic strategies interfering with non-native peptide and protein aggregation.

Computer simulation techniques have provided insight into several raised questions, such as thermodynamics or conformational dynamics of early oligomer formation [28]–[40] and additionally have made valuable mechanistic propositions [41]–[48].

In vitro studies established segments of amyloidogenic proteins and de novo designed peptides as suitable model systems to investigate sequence determinants of fibril formation (e.g. mutational effects) [49]–[54]. These short peptides were shown to be capable of forming amyloid-like fibrils, yet they are sufficiently small to allow systematic and controlled experimental access to their biophysical properties and to detailed structural models [55], [56]. Recently obtained crystal structures of such minimal peptide sequences provided insight into what could be the general spine organization of amyloid fibrils [57]–[61]. A common steric zipper motif was revealed for a number of peptides in the crystalline state, where two elongated sheets of peptide strands are arranged such that a complementarity packing of the side chains leads to a tight and dry interface. Combined, these experimental findings underscore the notion of amyloid fibril formation being a universal property of the peptide backbone depending on external factors and modulated by sequence characteristics [3], [62]. Moreover, it was shown that these specific short stretches can trigger self-assembly and mediate amyloid formation [63], [64], therefore leading to the idea that the amyloidogenicity of a sequence can be strongly localized. The ability of these segments to even force a globular, non-fibrillizing protein into the amyloid state was demonstrated [65].

In the present study we explore the structure and dynamics of spontaneously assembled oligomers of two different steric zipper peptides (PHF6, IB12) using atomistic molecular dynamics simulations in explicit water.

The PHF6 (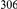
VQIVYK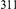) peptide is a segment from the microtubule-associated tau protein and has been shown to be sufficient for in vitro polymerization to filamentous structures and microcrystals [52], [58], [66], [67]. The PHF6 motif is located in the repeat regions of the microtubule-binding domain of tau and has been suggested to play a prominent role in the formation of paired helical filaments (PHFs) and is also part of the PHF core composed of cross-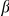 structure [66], [67], [9]. The natively unfolded and highly soluble full-length protein functions in assembly and stabilization of microtubules [67] and self-associates into PHFs, when hyperphosphorylated [9], [67]. Pathological accumulation of such tau aggregates into neurofibrillary tangles is a characteristic signature of Alzheimer's disease and other tauopathies [9].

The hexapeptide 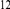
VEALYL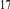, here referred to as IB12, is a segment from the B chain of the peptide hormone insulin and has been found to form amyloid-like fibrils, as well as microcrystalline aggregates with typical cross-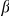 diffraction pattern [54], [58]. The IB12 peptide is proposed to have importance in full-length insulin misfolding and aggregation [68]. Insulin has been studied as a model system for aggregation and generally under conditions (elevated temperatures, low pH) which favor the monomeric and partially unfolded state [69]–[71]. Fibrillation proceeds via oligomeric, non-native intermediates, during which insulin is subject to major structural alterations from a predominantly 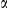-helical to 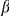-sheet rich conformation [8], [69]–[71]. According to several studies [58], [68], [70], [72] critical intermolecular interactions have been attributed to the IB12 segment of the B chain, which is also most likely incorporated in the extended 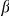-strands that make up the core region of insulin fibrils [73], [74].

Here we present a quantitative description of the spontaneous steric zipper peptide aggregation and oligomer growth process based on multiple submicrosecond molecular dynamics simulations. We use two different short segments of amyloidogenic proteins to study and unveil the critical variables that govern the kinetics of the initial biomolecular aggregation stages. From the observed conformational dynamics during the formation of small oligomers common mechanistic steps at molecular detail are elucidated. In order to do so, we introduce a novel low-dimensional mapping procedure to visualize the heterogeneous oligomerization pathways, allowing the identification of common encounter complexes and intermediates.

## Results

### Secondary structure and topology of spontaneously assembled steric zipper peptide oligomers

**Figure 1:**
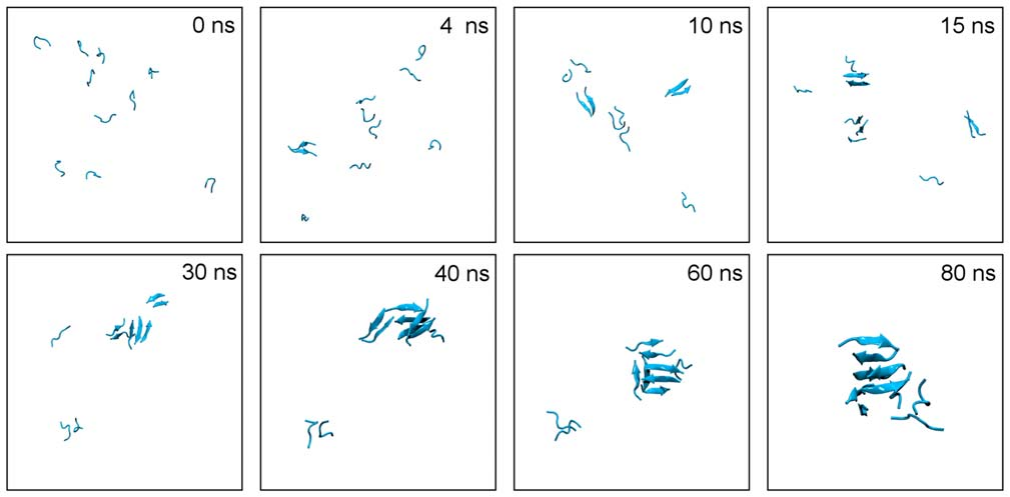
Illustration of primary PHF6 peptide aggregation events. Snapshots of conformations observed during the first 80 ns of a representative simulation of PHF6 oligomerization are shown (water and ions omitted for clarity). Starting from the initial state, the 10 monomeric peptides with random conformations rapidly assemble and start to form small sized aggregates (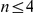) after a short lag time. Towards the end of the simulation an aggregated phase is observed, characterized by the presence of one single, large oligomer (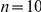).

In the present study we monitored the primary aggregation events for the two different peptides PHF6 and IB12, with eight independent simulations each (see Table 1). The simulations started from 10 separated peptide chains with random conformations, positions and orientations, respectively (Fig. S1). Representative snapshots from one of the PHF6 simulations are shown in Figure 1. They illustrate a typical, spontaneous self-assembly into 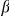-sheet oligomers as frequently observed in our 300 ns simulations and also visualized in Movie S1.

**Table 1:** Summary of performed simulations.

system (ID)	starting configuration	conc. (mM)	time (ns/run)	no. of sim.
VQIVYK (PHF6)	10 monomeric peptides, random	16.6	300	8
VEALYL (IB12)	10 monomeric peptides, random	16.6	300	8

conc.: peptide concentration.

no. of sim.: number of independent simulations.

**Figure 2:**
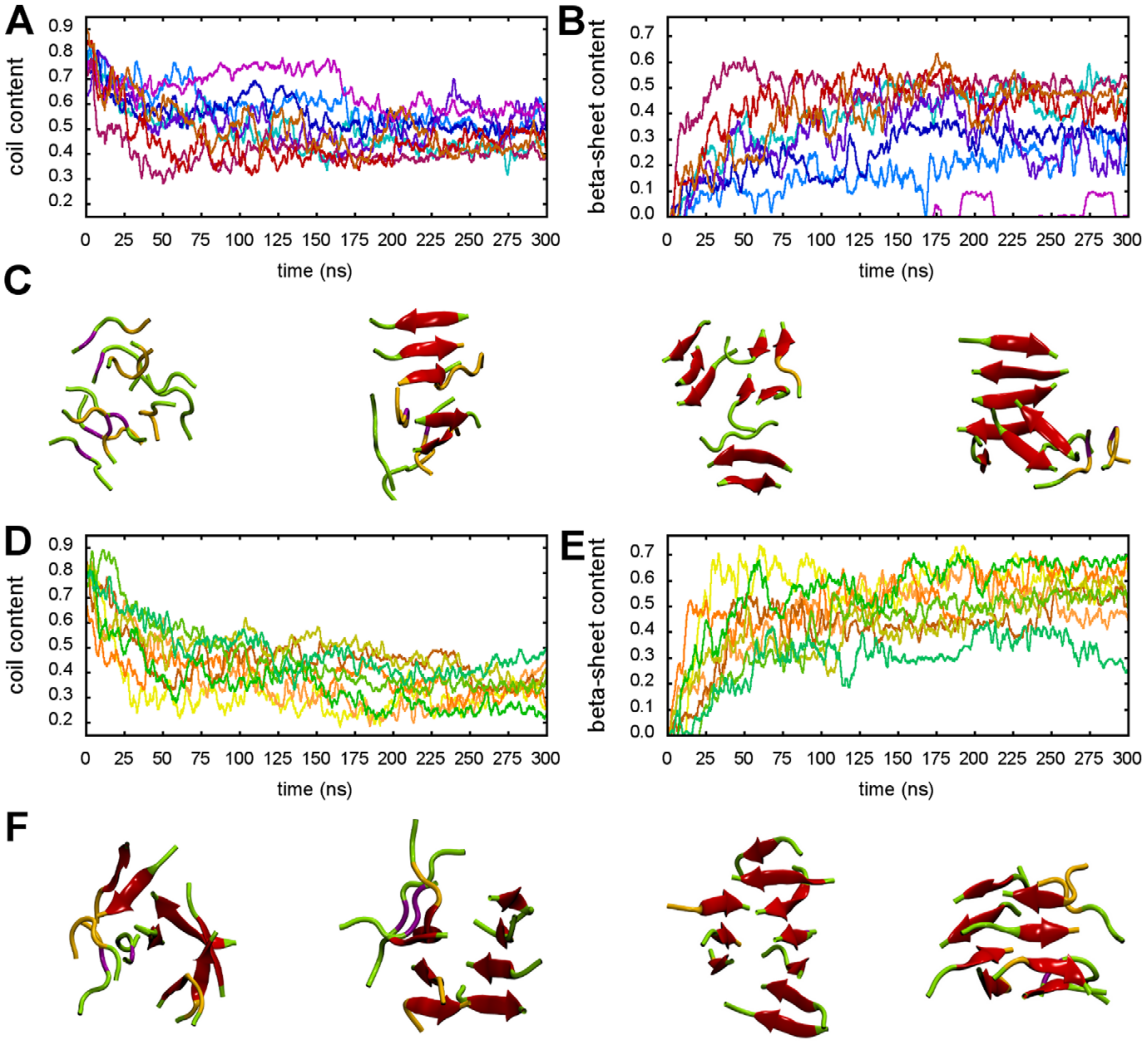
Analysis of the secondary structure evolution for PHF6 and IB12 oligomerization. The development of secondary structure elements according to DSSP is shown for all independent PHF6 (A, B) and IB12 (D, E) simulations. For PHF6 oligomerization a substantial conversion from random coil (A) to extended 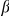-sheet (B) peptide structure is revealed. A set of representative PHF6 aggregate end-structures (300 ns) are depicted in cartoon representation, showing disordered, as well as intermediate and ordered 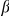-sheet assemblies consisting of several stacked 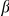-sheet subdomains (C). The colors encode 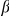-sheet (red), 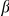-bridge (purple), random coil (green) and bend (yellow) secondary structure elements. The self-association of IB12 peptides and formation of larger aggregates was accompanied by an almost complete conversion from random coil (D) to extended 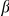-sheet (E) peptide structure in all simulation runs. A set of representative IB12 end-structures (300 ns) in cartoon representation shows 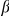-sheet rich oligomers, which were predominantly composed of bilayers of aligned 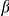-sheets. Barrel-like conformations with dry 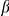-sheet interfaces were found to a less extent and exhibited residual disorder (F).

A secondary structure content analysis reveals that the peptide aggregation was accompanied by a conversion from random coil to extended 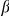-sheets as the dominant structural motif in the oligomeric state (Fig. 2). As simulation time progresses, a rapidly increasing number of inter-molecular hydrogen bonds was found, together with the spontaneous formation of 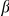-sheet rich oligomers. Although 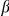-sheet formation was a general feature, differences in the 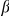-sheet content were observed for PHF6 and IB12 simulations. While in most of the PHF6 simulations more than 40% of the high initial random coil content was retained, for the IB12 peptides a lower coil fraction was found after 300 ns. In the course of the IB12 simulations a near monotonically increasing 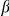-sheet content was observed (see Fig. 2E). On average more than half of all IB12 peptide residues were found in extended backbone conformation. In contrast, smaller fractions and larger fluctuations in the amount of 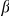-sheets were observed for the PHF6 aggregates, as shown in Figure 2B. One out of the eight PHF6 trajectories even yielded an almost amorphous decameric peptide aggregate, exhibiting strong disorder.

Nevertheless, the majority of all the observed aggregates did organize in 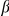-sheet rich structures, although topologically quite diverse (see Fig. 2C and 2F) when comparing individual simulations of the same sequence, as well as between the studied PHF6 and IB12 systems.

Among the most frequently sampled motifs in these heterogeneous structures were two or more opposing smaller 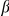-sheets stacked on top of each other. In addition, mostly single sheets of two to five strands facing a residual portion of disordered peptide chains were observed. With all PHF6 or IB12 peptides assembled into a single aggregate, 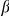-sheets with a perpendicular arrangement, as well as incomplete and distorted barrel-like orientations were formed transiently. A number of the spontaneously formed decameric peptide aggregates featured bilayers of well aligned and tightly laminated 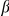-sheets. The resulting dry sheet interfaces constitute a structural characteristic of steric zipper cross-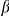 spines [58], although the observed oligomeric aggregates lacked the regular strand arrangement and complete side chain interdigitation as found in the crystalline conformation.

The described conformationally distinct oligomeric states with their relatively stable 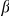-sheet rich subdomain conformations were found to interconvert between various forms of 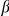-sheet aggregates. Indeed, most of the oligomers displayed orientational disorder and were rather dynamic due to an ongoing intra-sheet and inter-sheet side chain repacking. As a result 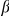-sheets were usually bent and twisted to some extent. Irregularly congregated sheets and weakly attached edge-strands were partially prone to break, shift, flip and reform during the simulations.

### Aggregate and 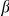-sheet size distributions

We followed the aggregate size distribution evolving during the series of eight independent trajectories per peptide system. In order to quantitatively probe the aggregation state of the peptides at any given time we pursued a hierarchical classification of the formed aggregates and their numerous conformations according to the criteria of general and 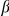-sheet peptide association (see Methods).

**Figure 3:**
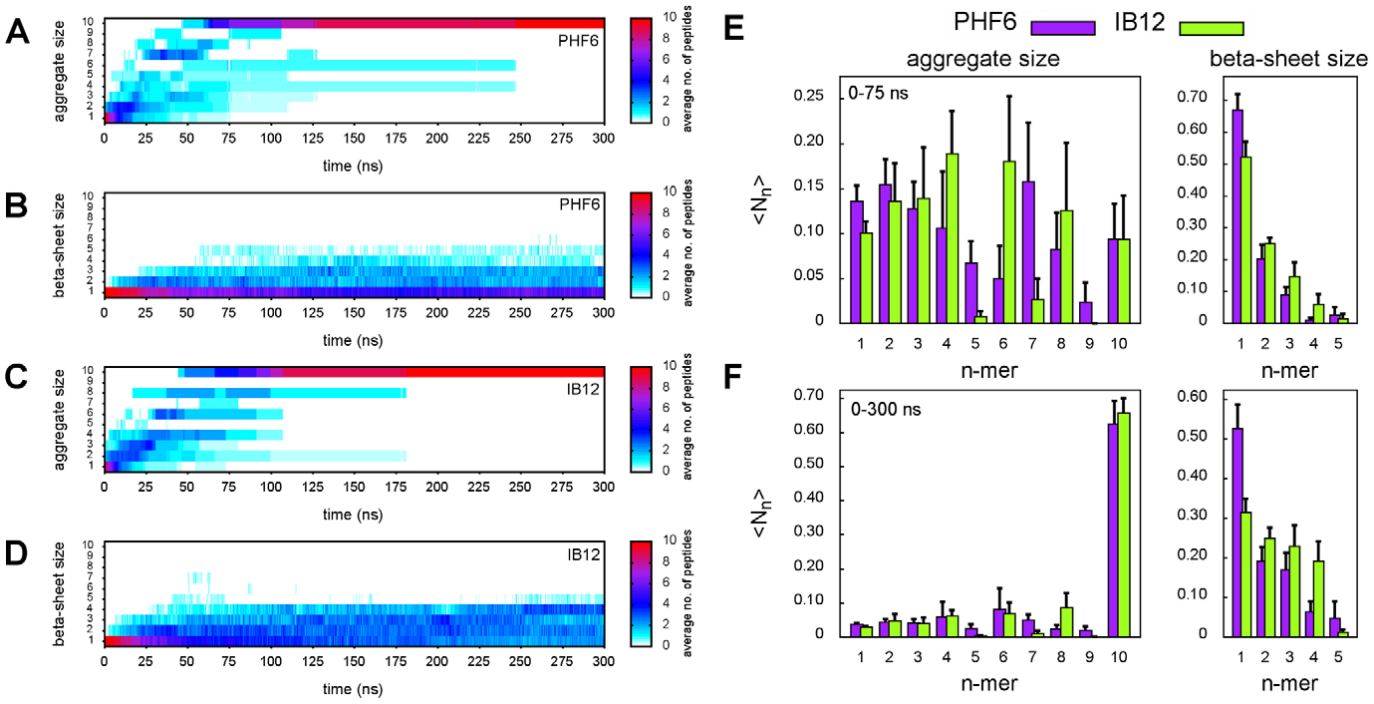
Time series and populations of aggregate and 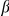-sheet size distributions. Averaged populations of general aggregate and 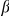-sheet size 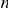 at simulation time 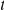 are shown for PHF6 (A, B) and IB12 (C, D), respectively. Dark-blue, purple and red colors indicate a high averaged abundance of a particular aggregate or 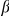-sheet size. The normalized aggregate and 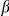-sheet size probability distributions for two time windows during the spontaneous aggregation simulations of both peptides are shown (E, F): from 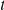 = 0–75 ns, which approximately corresponds to the early association and growth phase, after which the pool of monomers was found to be depleted in all simulations (E); for 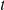 = 0–300 ns, where the distribution was dominated by the final aggregated phase and mainly characterized by a peptide reorganization process within the single, large aggregates. At this stage, stable but partially disordered oligomers with 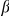-sheet subdomains of intermediate size emerged (F).

The averaged population of a specific aggregate size 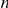 at a time 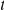 for the PHF6 and IB12 simulations is depicted in Figure 3A and 3C, respectively. The spontaneous oligomer formation was initiated by a rapid clustering of the peptides in all of the simulations. Starting from monomers, common association states were visited in the early phases of oligomerization. The peptide molecules were found to be dynamic and reversibly associating and dissociating, eventually ending up in the decameric state as indicated by the averaged occupations over all trajectories. The decamer represented a stable aggregation end-product in all simulations, as no substantial dissociation events of one or more peptides were observed.

The spontaneous peptide aggregation and oligomer formation process can therefore be characterized by two principal phases: an early association and growth phase where the assembly of peptides proceeded rather fast, and an aggregated phase where no further aggregation or disaggregation events took place, dominated by internal reorganizations.

Aggregates up to trimers were formed on average within a few nanoseconds (Fig. 3A and 3C). Higher order oligomers (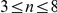) were formed within tens of nanoseconds. Interestingly, the IB12 peptide showed a rather uniform distribution of aggregate sizes over time (indicated by dark blue regions). In contrast, for PHF6 a broad and divergent distribution of association states was found. The aggregate populations of the individual simulations (Figs. S2 and S3) highlight the dynamical and complex assembly process into the decamers, suggesting that multiple assembly pathways exist.

From the computed normalized aggregate size probability distribution (Fig. 3E and 3F), we found that for PHF6 all possible oligomer sizes were populated at some point. For IB12, odd-numbered aggregate sizes larger than the trimer (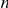 = 5, 7 and 9) were not or only briefly visited. Furthermore, the spectrum of IB12 peptide aggregate sizes in the association phase showed a preferential population of 4-, 6- or 8-mers compared to PHF6 simulations.

The early phase of peptide aggregation was most prominently marked by the burial of a large fraction of hydrophobic solvent accessible surface area (hSAS) in all performed simulation runs. Half of the initial hSAS was buried upon peptide self-association from monomers into multimeric assemblies and along with the formation of larger aggregates. This demonstrates that the initiation of peptide oligomerization was primarily associated with the reduction of nonpolar peptide surface, predominantly of the side chains. The hSAS values converged around 100 ns for both simulated peptide systems, respectively and displayed only little fluctuation afterwards (Fig. S5).

The onset of 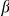-sheet formation (Fig. 3B and 3D) was slightly delayed in comparison to the general peptide assembly, suggesting that 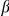-sheet formation was not the primary driving force for peptide aggregation. While dimeric or trimeric 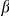-sheets formed relatively fast, noticeable build-up of 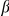-sheet assemblies composed of up to four or five 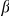-strands took place on time scales beyond 50 ns (Fig. 3E and 3F). However, the oligomers did not grow in one single sheet, as already discussed for representative snapshots from the simulations. They rather showed a tendency to be composed of at least two smaller sheets stacked on top of each other. The average size of these ordered subdomains was found to fluctuate strongly - consistent with frequent formation and breaking of backbone hydrogen bonds, even in the aggregate interior, reflecting the conformational plasticity of the observed oligomers (see also Fig. S2 and S3).

The aggregated phase started with the emergence of stable, but partially disordered decamers. All observed oligomeric end-states showed a low nonpolar surface area and compact arrangements, although the aggregates were still subject to structural fluctuations and reorganizations. This is in accordance with the overall higher abundance of three- and four-stranded 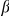-sheets (Fig. 3F), which was nearly twice as high compared to the initial stages of aggregate formation. While overall significant fractions of 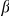-sheets composed of up to five strands were found, the propensity of sampling 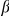-sheet sizes with two to four strands was on average higher in IB12 (2: 25%, 3: 23% and 4: 19%) versus PHF6 (19%, 17% and 6%) oligomers.

### Growth of aggregates proceeds via bimolecular association reactions

To further quantify and compare the spontaneous aggregation behavior of the PHF6 and IB12 peptide systems, we traced every individual association event in the simulations, starting in all cases from an ensemble of monomeric conformations. To exclude brief, unreactive collisions of peptide molecules from the analysis, we only considered association or dissociation of aggregates which were stable for at least 10 ps.

**Figure 4:**
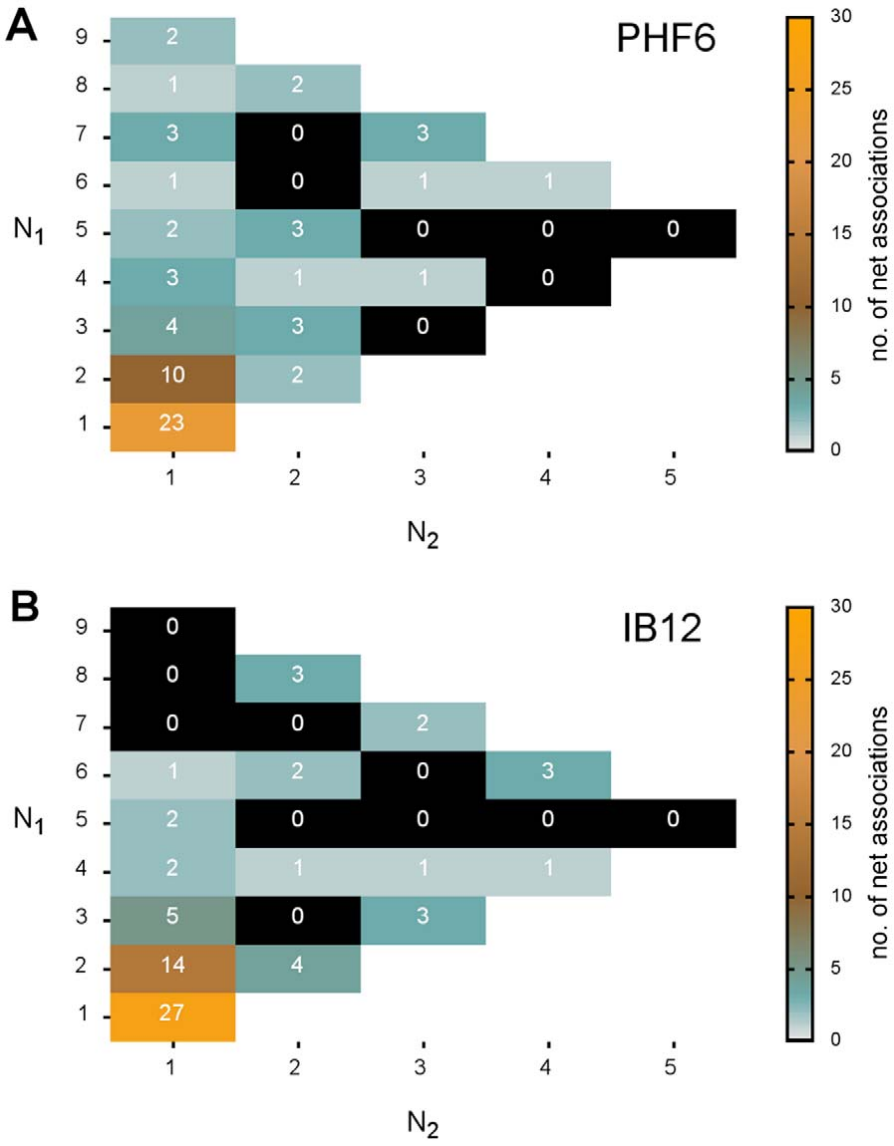
Oligomer formation and growth proceeded by bimolecular associations. The scheme highlights all possible bimolecular reactions up to the decamer (non-white boxes) and their respective number of occurrence in all of the PHF6 (A) and IB12 (B) simulations. The values in the boxes indicate the number of net association reactions between aggregates of size 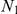 and 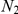 in the general form: 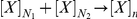; 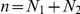; 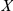 = PHF6, IB12 (i.e. the overall productive oligomer formation and growth steps).

We found that oligomer formation and growth proceeded exclusively by bimolecular association reactions, no trimolecular or higher order association reactions were observed. The observed association events thus can be represented in the general form:
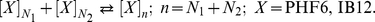



The net associations observed in all PHF6 and IB12 simulations are summarized in Figure 4A and B, respectively.

After the initiation step of the pairing of two monomers and irrespective of the investigated sequence, aggregate growth was found to proceed heterogeneously, as already seen in the series of time dependent oligomer size distributions (Fig. 3A and 3C).

However, we could distinguish two principal reaction types according to basic kinetic models of non-native protein aggregation [18]: aggregate growth by (chain) polymerization and condensation. Polymerization type reactions cover additions of one or more monomers, whereas condensation reactions involve any other aggregate-aggregate association step that does not directly consume monomers.

In the case of PHF6, decameric oligomers and aggregates of all sizes were found to grow by parallel routes, either by adding one monomer at a time (first column in Fig. 4A; 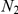 = 1) or through condensation reactions (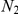 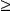
2). For the IB12 assembly we observed a less diverse set of bimolecular association reactions and more of a multi-staged process. Here, the mutual fusion of aggregates (condensation) was the dominant pathway for the formation of larger aggregates. Especially dimeric or trimeric IB12 aggregates were found to condense preferably, as well as dimers with tetra-, hexa-, and octamers. These prevalent association reactions were also reflected in the marginal population of odd-numbered general aggregate sizes for IB12, in contrast to the PHF6 simulations (Fig. 3E).

Moreover, smaller fluctuations and a larger irreversibility was found for IB12 peptide association in comparison to PHF6, when examining all the individual association and dissociation events (see Fig. S4). We found that the efficient assembly into dimers and trimers leads to a fast and irreversible depletion of available IB12 monomers. This offers a plausible explanation why IB12 aggregates larger than heptamers exclusively were found to grow by condensation type reactions. PHF6 peptides were found to associate less efficiently or to dissociate after transient contact formation, which gave rise to more complex assembly pathways all the way to the decamer.

### Conformational properties of oligomeric aggregates with various size

**Figure 5:**
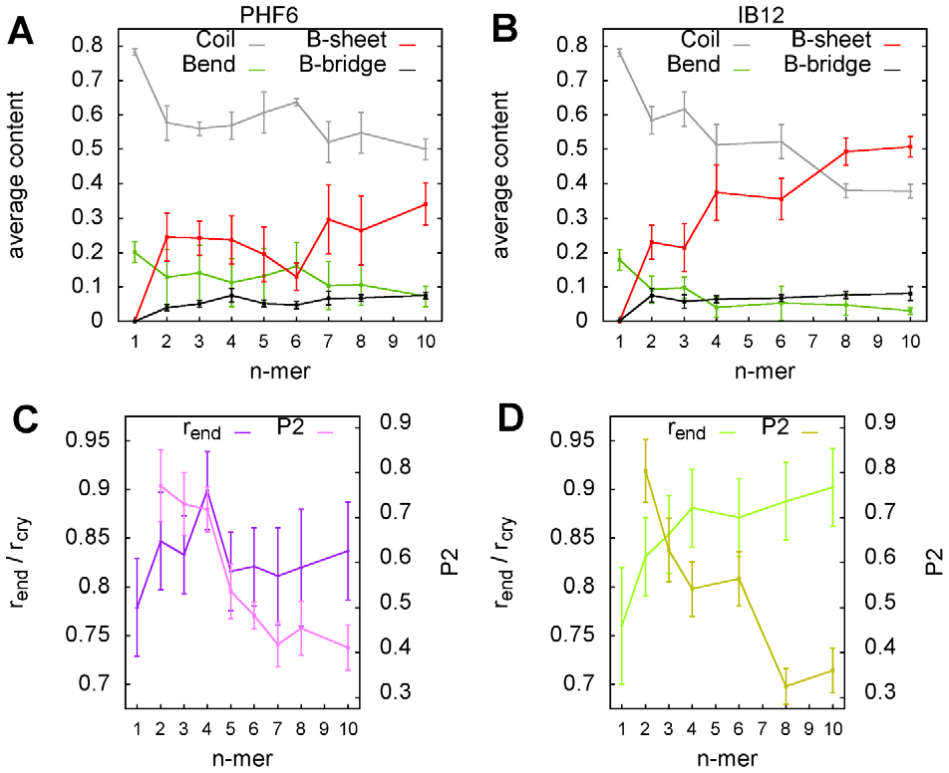
Structural analysis of PHF6 and IB12 oligomers as a function of the number of peptides contained. Secondary structure propensity according to DSSP based on all simulations for PHF6 (A) and IB12 (B) aggregates. An increase in 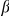-sheet structure with aggregate order 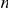 was observed for both peptide systems. However, the secondary structure content observed for aggregates larger than trimers differed significantly for PHF6 with respect to IB12. The average intra peptide distance between the N- and C-terminal C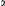-atoms and the nematic order P2 is shown for PHF6 (C) and IB12 (D) oligomers. The calculated peptide chain extension (r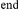) was normalized to the respective distance in the conformation found in the crystal structure (r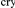). The analysis reveals that the individual PHF6 and IB12 peptide chains extended with the order of the aggregate 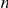 (C and D). The nematic order of the aggregates decreased generally with size. Large PHF6 oligomers (C) had on average a higher orientational order compared to IB12 aggregates (D) of the same size, yet they aggregated in less extended chain conformations.

In order to further access the molecular details of assembly, as well as aggregate structure and dynamics, we examined several essential features of the spontaneously formed oligomers as a function of aggregate order 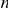 (Fig. 5). Our analysis was based on all performed simulation runs, but we excluded oligomer sizes for which statistics were insufficient (less than 50 ns of cumulative occurrence; PHF6: 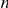 = 9; IB12: 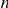 = 5, 7 and 9).

First of all, certain conformational quantities seem to depend on the aggregate order, but to a different extent for both investigated peptide systems. This can be seen for the formation of secondary structure elements as shown in Figure 5A and 5B. No significant secondary structure was found for the peptide monomers, as they adopted predominantly coiled (roughly 80%) and bend conformations regardless of the sequence. Starting with dimers we observed a significant conversion from coil to 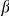-sheet structure. While PHF6 oligomers of intermediate size maintained the average 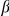-sheet content of the dimeric aggregates (around 25%), a marked increase in 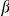-sheet conformations from IB12 dimers to trimers and tetramers was detected. The decameric aggregates exhibit the highest 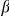-sheet content among all aggregate sizes of both studied peptide sequences. However, the IB12 decamers showed a higher and more uniform amount of 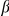-sheet structure in comparison to PHF6 assemblies of this particular size.

In terms of structural content per residue as assigned by DSSP [75], we found that for both peptides the central hydrophobic aliphatic residues Ile3 (PHF6) and Ala3 (IB12), as well as the respective flanking residues have the highest probability to adopt 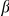-sheet conformation. This feature was conserved from the dimers to the decamers. Interestingly, PHF6 and IB12 peptides sampled extended conformations at position five less frequently, which is in both cases a tyrosine residue.

We found that the end-to-end distances of the peptides were shifted gradually towards the ones of more stretched conformers, as the individual strands organized in larger aggregates (Fig. 5C and 5D). Especially the IB12 peptide chains were driven to extended conformations due to the presence of inter-peptide interactions, along with the appreciable change in secondary structure. This feature was found to be less distinct for PHF6 peptides, consistent with the persistent sampling of random coil and bend structures.

To asses the alignment order of specific aggregates, we use the nematic order parameter P2, which discriminates between uniaxial, ordered or disordered (amorphous) conformations (see Methods). Here P2 values larger than 0.5 indicate the propensity to be in an ordered, well aligned state. Figures 5C and 5D show the averaged nematic order of aggregates with size 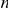. The initially very high orientational order in the spontaneously formed peptide dimers (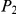 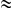
0.8) decreased prominently for IB12 trimers and tetramers (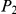 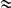
0.55–0.6), whereas PHF6 tetramers still exhibited high nematic order. The averaged orientational order was lowest in the IB12 decamers (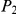 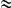
0.35) despite the fact that these aggregates contained the highest amount of 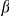-sheet structure and sampled highly extended conformations (Fig. 5D). This can be attributed on one hand to the strong fluctuations due to conformational reorganizations present in all of the aggregates. On the other hand this is due to the degree of disorder caused by lateral stacking and twisting in the larger 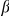-sheet assemblies.

A mixture of parallel and anti-parallel peptide strand alignments was found within 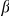-sheets of all sizes for both, PHF6 and IB12 aggregates. Strands aligned preferably anti-parallel in IB12 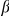-sheet dimers, whereas parallel orientations were more prominent in larger IB12 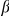-sheet aggregates. A general preference for anti-parallel orientations was persistently found for PHF6 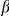-sheets of all observed sizes. The patterns of peptide strand registry were found to be heterogeneous in both peptide systems.

### PHF6 and IB12 peptides form anti-parallel 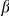-sheet dimers

As seen from the secondary structure analysis, peptide dimers were critical intermediates in PHF6 and IB12 oligomerization. This particular observation was made with regard to the significant conversion from random coil to 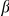-sheet structure. Furthermore, peptide dimerization was the primary step of the aggregation process. It is therefore of interest to investigate the influence of the specific dimer conformation on the formation of larger oligomeric structures. We do this by analyzing the relevant participation of dimers in the discussed reaction types and association pathways. A detailed analysis of the conformational characteristics and dynamics of the dimeric aggregates was therefore carried out.

**Figure 6:**
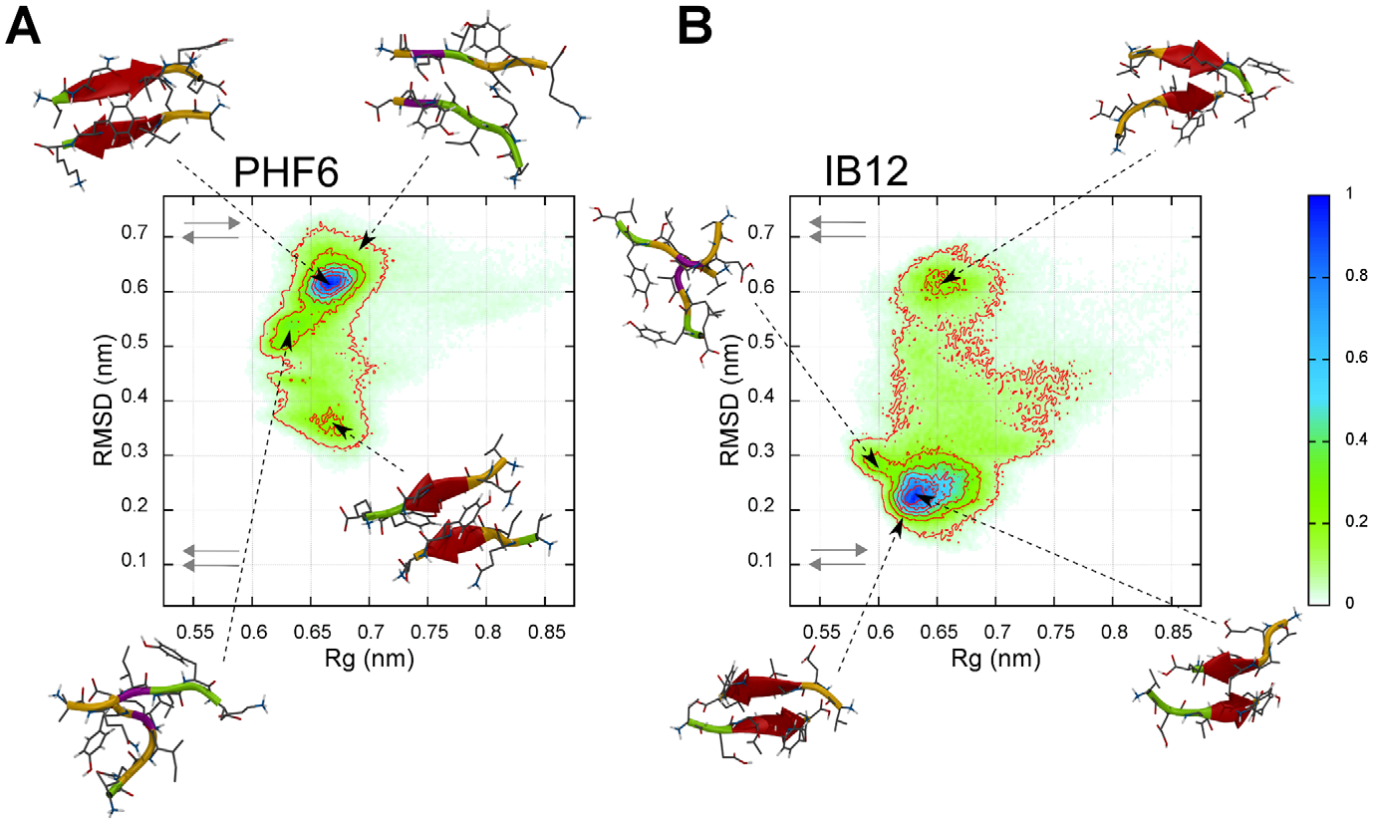
Ensemble of spontaneously formed PHF6 and IB12 peptide dimers. A projection of the spontaneously formed dimer ensemble as a function of radius of gyration (R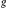) and the C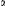 root-mean-square deviation (RMSD) to the known crystal structure conformation of the PHF6 and IB12 peptides (2ON9, 2OMQ) is depicted, respectively. Corresponding alignment states are schematically highlighted by gray arrows in the upper and lower left part of the projections. Representative structures for peptide dimers with a normalized probability higher than 0.3 are shown for PHF6 (left) and IB12 (right) dimers. The normalized frequency of occurence scale is given on the right.

A projection of the spontaneously formed PHF6 and IB12 dimers collected from all simulations onto two observables (R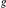, RMSD) is shown in Figure 6 together with representative structures of frequently visited conformational states. Overall, both ensembles display a similar landscape featuring a variety of well-aligned anti-parallel, less ordered parallel, orthogonal, compact as well as largely unstructured chain conformations.

From the normalized sampling probability for PHF6 dimers in Figure 6A we found that one major and several minor dimer conformations were populated. The statistically most significant structure was an ordered, anti-parallel 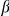-sheet, which deviates 0.62 nm in RMSD from the known parallel crystal structure arrangement. Another frequently sampled conformation did not have extended 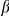-strands, but rather showed disordered, but compact peptide chains, which were stabilized by an isolated 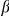-bridge (0.53 nm RMSD). Parallel, out-of-register 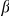-sheet dimers of intermediate order appear at 0.375 nm RMSD. Parallel, in-register dimers were not formed spontaneously.

Figure 6B shows the projection of the spontaneously formed IB12 dimers. We found an anti-parallel 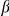-sheet with out-of-register conformation (0.22 nm RMSD) as the most abundant structure. A collapsed, disordered state (0.3 nm RMSD) stabilized by a single backbone hydrogen bond pair and contacts of the N-terminal side chains were also found with a high probability. Disordered or partially ordered, parallel 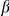-sheet structures were sampled as well, but to a smaller extent. A significant portion of less compact structures with a large radius of gyration was seen in comparison to PHF6 dimers.

A notable finding was the prevalent anti-parallel strand alignment for the dimer conformations of both peptide sequences. The alignment corresponded well to the native filament pattern of the IB12 crystal structure. However, the spontaneous aggregation simulations of the PHF6 peptides did not sample the formation of ordered, parallel dimers, which approached the respective crystalline reference state with a RMSD of less than 0.3 nm.

To systematically test the stability of the fibril-like dimer peptide arrangement we ran a total of 20 short simulations (15 ns) of isolated dimers per peptide sequence (see Text S1). This particularly addressed the question whether the parallel PHF6 dimer was structurally stable on the nanosecond time scale. Two exemplary simulations were prolonged up to 1 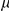
s to probe the dimer dynamics beyond the nanosecond timescale.

We chose two preformed 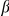-sheets in an ordered, either parallel or anti-parallel arrangement as initial conformations and representative dimeric states. One of them was the experimentally determined crystal structure conformation, the other one was an ordered 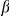-sheet dimer extracted from the spontaneous aggregation simulations with opposite strand polarity.

In the simulations of isolated, parallel PHF6 dimers we saw an apparent trend towards larger RMSD values with respect to the x-ray reference structure. The increase in RMSD was the result of thermal fluctuations and a twist in the 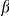-sheet (similar to the parallel, out-of-register structure in Fig. 6A), which differed from the untwisted crystalline conformation by up to 0.275 nm. The ordered parallel alignment of the 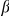-sheet strands was likely destabilized by electrostatic repulsion of the like charges at the peptide termini and also resulted in a decrease of 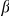-sheet structure.

Transitions from parallel to anti-parallel PHF6 dimer conformations were observed only once in the 10 short simulations. Including also the interconversions observed in the extended simulations, we can calculate a rate constant of 3.9

10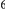 per s. A dissociation of the preformed dimer aggregates did not occur.

We took that same approach to examine IB12 dimers with parallel or anti-parallel starting structure. Here, we found that the anti-parallel IB12 reference was not stable beyond 25 ns in the extended simulations and explored less compact, anti-parallel out-of-register conformations (similar to the representative structure highlighted in Fig. 6B), as well as parallel dimer structures. The total number of interconversions from parallel and anti-parallel conformations corresponds to a rate constant of 2.2

10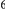 per s.

In summary, the ensembles of spontaneously formed PHF6 and IB12 dimer structures were relatively heterogeneous with predominantly anti-parallel chain conformations. However, from multiple validation simulations of isolated dimer structures we found that parallel PHF6 and IB12 dimers are stable as well. For the isolated anti-parallel IB12 reference dimer structure only a limited kinetic stability was revealed.

The preformed PHF6 and IB12 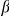-sheet dimer conformations can inter-convert between different alignment states on the sub-microsecond timescale, which is an order of magnitude longer than the average life time of dimers in the spontaneous aggregation simulations. Although the calculated rates for interconversion of dimer alignments should be taken with care considering the potential bias of starting structures and limited statistics in our simulations, the associated barrier heights can be estimated to be approximately 30 kJ/mol, for both PHF6 and IB12 applying rate theory and assuming an attempt frequency of 1 per ps.

In addition to simulations with GROMOS96 43A1, we included the AMBER99SB and CHARMM27 force fields to test the structural properties of preformed and encountering PHF6 and IB12 dimer aggregates (Text S1). All these force fields are frequently used and have been shown to perform particularly well in peptide aggregation and folding simulations [35], [42], [76]. The different molecular mechanics force fields were compared in their ability to characterize and preserve the isolated dimer structures (Text S1). To that end way we evaluated and validated our findings for the simulations of spontaneous peptide aggregation with a consensus force field approach [76]. We found that the aforementioned twisting of parallel PHF6 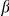-sheet dimers appears to be slightly less prominent when simulating with the CHARMM27 force field compared to the GROMOS and AMBER force fields. In the simulations of preformed, anti-parallel PHF6 dimers, as well as parallel IB12 dimers, we observed a similar behavior and stability in all the tested force field variants. Therefore it appears that the choice of force field is only a minor concern with respect to stability of preformed 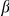-sheets.

### Conformational mapping on collective coordinates yields distinct conformational states of PHF6 and IB12 dimers

To address the question how the conformational dynamics of the peptide dimerization plays a role in the selective population of the anti-parallel conformations and how this can translate to structural features of larger aggregates, we introduce a novel method of conformational mapping (see Methods).

With a principal component analysis (PCA) we obtained collective coordinates to describe a peptide aggregate by means of its large-scale structural fluctuations in a space of reduced dimensionality. We thereby determine the conformational changes associated to, for example initial peptide encounter complexes, but also conformational transitions within formed aggregates. To be able to map multimeric aggregates larger than dimers we describe oligomeric structures in terms of minimal, dimeric units. The application of a clustering procedure then allows for a consistent description of conformational distributions and association modes. With this approach we address a fundamental challenge, which is the determination of a way to analyze the structural aspects of the large and manifold ensemble of multimeric aggregate conformations sampled in our simulations.

**Figure 7:**
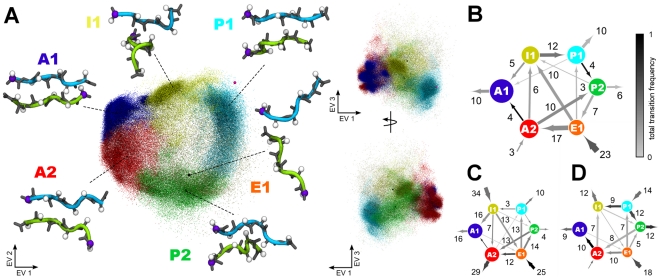
Mapping of PHF6 aggregate sizes and transitions between individual conformational clusters. On the left side of the panel: Visualization of PHF6 dimer conformations in a three-dimensional principal subspace of collective fluctuations (A). Each dot corresponds to a structure of the simulation projected on the first three eigenvectors as obtained from a principal component analysis. The projection is viewed from three different angles. The colors indicate the assignment of a PHF6 dimer structure to one of six conformational states: A1, A2, P1, P2, I1, E1. The states were labeled with a capital letter according to the overall dimer aggrangement within the respective cluster: A - anti-parallel dimer; P - parallel dimer; I - intermediate ordered or irregular dimer structure, E - encounter complex. Cluster centers are represented as black spheres, parallel and anti-parallel reference dimer conformations are highlighted as magenta spheres. The center structures from each cluster are shown as main-chain (gray sticks) and C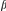 atoms (white spheres), as well as the backbone in a cartoon representation. The N-terminal C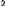 atom of each chain is shown in purple. On the right side of the panel: Transition networks illustrate the net transitions between the clusters as observed for dimers (B), trimers (C) and tetramers (D). The node colors match the clusters in the projection, the node size is consistent with the total number of assigned structures. The width and direction of the node edges corresponds to the number of net transitions. The total transition frequency between two nodes is color coded according to the gray scale on the top right corner.

**Figure 8:**
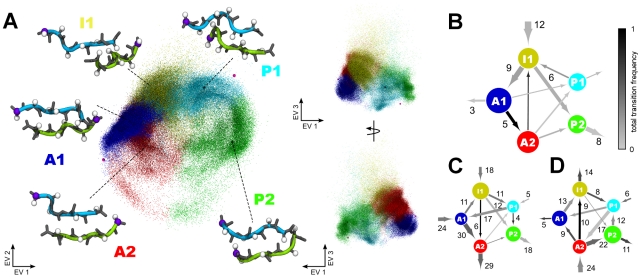
Mapping of IB12 aggregate sizes and transitions between individual conformational clusters. On the left side of the panel: Visualization of IB12 dimer conformations in a three-dimensional principal subspace of collective fluctuations (A). Each dot corresponds to a structure of the simulation projected on the first three eigenvectors as obtained from a principal component analysis. The projection is viewed from three different angles. The colors indicate the assignment of an IB12 dimer structure to one of five conformational states: A1, A2, P1, P2, I1. The states were labeled with a capital letter according to the overall dimer aggrangement within the respective cluster: A - anti-parallel dimer; P - parallel dimer; I - intermediate ordered or irregular dimer structure. Cluster centers are represented as black spheres, parallel and anti-parallel reference dimer conformations are highlighted as magenta spheres. The center structures from each cluster are shown as main-chain (gray sticks) and C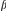 atoms (white spheres), as well as the backbone in a cartoon representation. The N-terminal C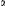 atom of each chain is shown in purple. On the right side of the panel: Transition networks illustrate the net transitions between the clusters as observed for dimers (B), trimers (C) and tetramers (D). The node colors match the clusters in the projection, the node size is consistent with the total number of assigned structures. The width and direction of the node edges corresponds to the number of net transitions. The total transition frequency between two nodes is color coded according to the gray scale on the top right corner.

First, we focus on the main structural features of the dimeric states as revealed by the PCA and the conformational clustering. Figures 7A and 8A show a three-dimensional representation of the conformational space of the spontaneously formed PHF6 and IB12 peptide dimers, projected onto the first three principal components - eigenvectors - EV1, EV2 and EV3, respectively. The conformational clusters were labeled according to definitions given in Figures 7 and 8, respectively.

Surprisingly, the obtained collective coordinates of the PHF6 and IB12 dimer structure ensembles were found to be similar. From both the projections, one can identify anti-parallel (cluster A1 and A2) and parallel (cluster P1 and P2) dimer conformations, which are discriminated along EV1. A twisting mode is described by EV2 and alters the relative chain orientations, respectively.

In the case of the mapped PHF6 dimers (Fig. 7A) several dense regions in cluster A1 and A2, as well as P1 and I1 were evident. Interestingly, the center structures of cluster A1, I1 and P1 correspond well to the representative ordered anti-parallel, partially ordered parallel and disordered structures in Figure 6A. Especially sparse and diffuse were the mapped structures along EV3 in the upper part of both projections. Here, the center of mass distance between the two peptide chains was large, and indeed a chain separation mode was the one mostly described by the third eigenvector. It can also be seen that for PHF6 around the reference parallel dimer (magenta sphere in the upper right of Figure 7A) no sampling density exists. For the mapped conformational space of the IB12 peptide dimers (Fig. 8A), an important difference to PHF6 was the identification of only five clusters as compared to six found for PHF6. Furthermore, we saw less symmetry with respect to the mapping of the IB12 structures along EV2. Again, cluster and densely populated regions matched the prominently sampled states in Figure 6B (A1, A2, P1, I1).

In the spontaneous aggregation of IB12 peptides neither fully ordered parallel nor anti-parallel in-register structures were sampled (magenta spheres in Fig. 8A).

Averaged structural features of the spontaneously formed dimers were extracted based on the conformational clustering and are summarized in Tables 2 and 3. To compare the distinct structural properties of the clustered aggregate structures to one another, we calculated the hydrogen bond energy according to Espinosa *et al.* [77] and decomposed it into several contributions. In addition, we analyzed the hydrophobic solvent accessible surface, radius of gyration and average number of residues in 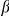-sheet conformation for each cluster.

**Table 2:** Averaged structural features of the conformational clusters of the PHF6 dimers.

cluster	f	N 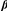	hbE 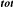	hbE 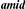	hbE 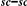	hbE 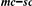	hSAS	R 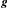
A1	0.34	2.4	−104.72	−80.26	−2.69	−6.26	9.63	0.671
P1	0.15	1.1	−78.54	−52.40	−3.61	−7.48	9.90	0.667
P2	0.13	1.1	−68.21	−46.94	−1.99	−7.55	9.99	0.668
I1	0.14	1.1	−79.60	−54.46	−1.70	−5.84	9.83	0.655
E1	0.07	1.0	−63.30	−40.80	−1.81	−7.27	10.96	0.832
A2	0.17	0.7	−64.02	−37.78	−1.33	−7.77	10.07	0.677

f: Fraction of dimer structures assigned to this conformational cluster.

N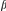: Number of residues in 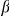-sheet conformation (per chain).

hbE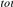: Total hydrogen-bond energy of the dimer complex (kJ/mol).

hbE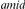: NH - CO backbone hydrogen-bond energy (kJ/mol).

hbE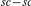: side chain - side chain hydrogen-bond energy (kJ/mol).

hbE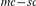: main chain - side chain hydrogen-bond energy (kJ/mol).

hSAS: hydrophobic solvent accessible surface area (nm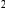).

R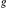: Raduis of gyration (nm).

**Table 3:** Averaged structural features of the conformational clusters of the IB12 dimers.

cluster	f	N 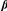	hbE 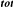	hbE 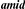	hbE 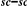	hbE 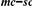	hSAS	R 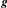
A1	0.35	2.1	−99.75	−85.55	−0.76	−1.07	9.87	0.644
P1	0.10	0.9	−59.62	−47.42	−0.64	−2.96	10.20	0.674
P2	0.19	0.5	−67.32	−51.70	−0.20	−2.16	10.36	0.718
I1	0.12	1.4	−70.77	−58.26	−0.28	−4.44	10.11	0.663
A2	0.24	1.2	−77.87	−67.35	−0.61	−2.20	10.13	0.660

f: Fraction of dimer structures assigned to this conformational cluster.

N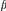: Number of residues in 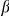-sheet conformation (per chain).

hbE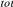: Total hydrogen-bond energy of the dimer complex (kJ/mol).

hbE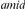: NH - CO backbone hydrogen-bond energy (kJ/mol).

hbE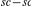: side chain - side chain hydrogen-bond energy (kJ/mol).

hbE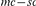: main chain - side chain hydrogen-bond energy (kJ/mol).

hSAS: hydrophobic solvent accessible surface area (nm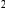).

R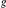: Raduis of gyration (nm).

The cluster A1 was the most populated state for the two studied peptides. A1 has the highest amount of 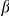-sheet, the lowest total hydrogen bond energy for the dimer complex and also the most reduced hydrophobic surface area, respectively. Inter side chain hydrogen bonds are negligible in the case of IB12 dimers, while there were substantial energetic contributions for PHF6, especially in the parallel dimers of cluster P1.

To obtain the extent and pattern of inter-peptide contact formation present in the individual conformational clusters, we calculated the average probability of inter-peptide residue pair contacts. This was achieved by averaging over all the structures of a cluster, respectively. The color-coded contact maps are shown in Figures S6 and S7 and reflect the overall arrangement of the peptide chains (alignment, registry) in the respective conformational cluster. We found that the central hydrophobic residues formed contacts with high probability, both in PHF6 (Ile3, Val4) and IB12 (Ala3, Leu4) dimers. Interestingly, the averaged contact maps for the individual IB12 dimer clusters show that the hydrophobic C-terminus (Leu6) was involved in many interchain contacts. In the case of PHF6 dimers, fewer contacts were observed involving the C-terminal end, while the Gln2 residue was found to participate prominently in contact formation (e.g. in cluster A2, P2 and E1).

### Kinetics of dimerization are sequence dependent

The obtained ensemble of spontaneously formed dimer structures cover every step from diffusional encounter of two peptide molecules to primary contact formation and conformational reorganization of the initially formed aggregates. In order to analyze the kinetics of dimerization in more detail, we constructed transition networks between the identified clusters of peptide conformations (see Methods). We thereby gain mechanistic insight into common and alternative pathways. Here, we focused on the pathway analysis of assembly into dimers, trimers and tetramers (Fig. 7B–D and 8B–D), as these aggregates were involved in the primary aggregation stage, covered most of the initial association steps and yielded adequate statistics.

First of all we focus on the respective dimerization events. From Figure 7B we see that encountering PHF6 peptides frequently attached loosely or transiently via structures similar to E1. This was predominantly facilitated by the contact formation of the N-terminal Gln2 residue of one peptide to the C-terminal Lys6 of the other (Fig. S6). The dimeric aggregates from this generic encounter complex ensemble (E1) were, however, not stable and evolved further via two main pathways. We identified either a sequential zipping up of the extended peptides to result in A2 type structures or a collapse of the peptide chains towards strongly disordered I1 conformations. Here, the compaction mainly originated from the burial of the central hydrophobic parts (Ile3, Val4). Access to the ordered anti-parallel dimer conformations of cluster A1 was found to be possible from both of these configuration types and accompanied by increasing backbone hydrogen bond interactions (see Table 2). However, dimer structures from the I1 cluster were found to reorganize predominantly to parallel dimers (P1, P2).

The IB12 dimer ensemble lacks a discrete encounter complex cluster with well separated peptide chains, as can be seen in Figure 8B. A common first conformational state of peptide association exists in the disordered, but compact cluster I1 type structures, which were stabilized by packing interactions of the hydrophobic C-terminal Leu4, Tyr5 and Leu6 residues (Fig. S7). From this pool of conformations transitions to ordered conformations in the large cluster A1 and out-of-register A2 dimers occurred, as well as to the P2 cluster. A direct interconversion of P1 and P2 structures was not seen, while structure transitions between clusters A1 and A2 were observed with a high frequency.

To summarize the observations made for the dimers so far, the pathway analysis indicates that the peptide dimerization is facilitated through the formation of specific key residue contacts between the two strands, respectively. From less directional, early encounter conformations a general ordering transition with increased backbone hydrogen bond interactions was observed. Both peptide systems differed in the dynamics and complexity of association pathways. For IB12 peptides the initial hydrophobic collapse brought the peptide chains together rapidly. These less ordered dimer conformations are characterized by a large number of mutual contacts and a significant portion of backbone hydrogen bonds. From there a reorganization generally took place that further maximized the inter-peptide interactions. In the case of PHF6, the observed dominant association pathway started from a generic encounter complex with specific side chain contacts. Here, a sequential gain of interactions was found, where residue contacts and backbone hydrogen bonds formed throughout the molecules in a zipper-like fashion. In addition, a less ordered collapse of peptide chains prior to conformational rearrangement was found.

### Dimeric versus multimeric conformational dynamics

We probed the trimer and tetramer ensembles through the perspective of dimeric structures to asses the changes to the dynamical behavior of these higher order oligomers with respect to the actual dimers (see Methods).

Here, more complex dynamics were found, reflected in the different transition patterns among the conformational cluster types, as well as in and out of the network (Fig. 7C–D and 8C–D). In the following we expand on a number of mechanistically relevant findings obtained from these complex transition networks.

For PHF6 we found that the pathway identified for the dimerization (from cluster E1 to anti-parallel conformers) persisted for trimers and tetramers. Transitions from E1 to parallel structures (P1, P2) were now seen as well. In particular P2 type conformations were often found to be prone to rearrange to A2. Ordered anti-parallel dimers (A1, A2) were the largest conformational ensemble and also dominant end-conformations (exit nodes). However, the described conformational reorganization and ordering transitions were slower or less often sampled. Evidence comes from a stall at the intermediate locked and partially zipped A2 structures in the trimer and the collapsed and disordered structures (I1), which were mainly found as the initial or end-conformations.

In the case of IB12 trimer and tetramer assembly dynamics mainly involved an ordered, preformed complex (A1), a seed structure onto which incoming peptides collapsed and added up to in a parallel fashion. The preformed 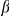-sheet dimers acted as suitable templates and parallel 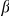-sheets formed easily in many instances. This demonstrates a directional strand assembly different from the dimerization case. The P2 and P1 clusters were the largest sampled clusters for the aggregates of order 3 and 4. In the process of accommodating a free monomer or dimer, the preformed structured oligomer underwent large fluctuations, which is linked to orientational disordering of the individual peptides (see Fig. 5B). This is in line with the sizable population of the cluster A2, P2 and especially I1. In contrast to the PHF6, rearrangement from parallel to anti-parallel IB12 alignment states was found and did not involve intermediate structures of type A2 or I1. This pathway was especially frequently seen in the tetramer. Similarly, we found reptation out-of-register transitions to go from A1 to A2. This suggests that the possibility to transit among different conformation types within an aggregate was dependent on the oligomer size and characteristics. The increased internal fluctuations of the aggregates and flexibility of the peptide molecules was more pronounced for IB12 compared to PHF6. A likely reason is the larger hydrophobic patch in the IB12 sequence, and in turn the larger influence of weak dispersion interactions on aggregate energetics and dynamics.

## Discussion

### Oligomer structure and dynamics of association and reorganization are heterogeneous

The observed early oligomers were found to be partially ordered structures rather than completely extended peptide chain conformations [28], [33], [35], [45], [78], [79] and lack a uniform strand registry and alignment. We observed densely packed oligomers with residual orientational disorder, but predominantly high 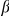-sheet content. Although topologically quite diverse and heterogeneous structures emerged from the simulations after 300 ns, some of these aggregates displayed structural characteristics of the crystalline conformation, namely stacked 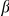-sheet bilayers with steric zipper-like interfaces [57], [58].

The aggregates were found to grow via multiple and diverse bimolecular association reactions. Our work demonstrates that the formed aggregates are dynamic and undergo substantial conformational reorganization during the growth and accommodation of monomers and small intermediates. Moreover, the characterization of multichain configurations by decomposition and mapping of dimeric structures reveal differences in conformational ensembles of oligomers of different orders and an assembly process that was heterogeneous at the molecular level [40], [80]. This indicates that association and reorganization pathways are dependent on the oligomer size and characteristics.

The interconversions between many distinct oligomeric states after the growth stage occurred without full or partial detachment of the peptide strands from the aggregate. We found multiple internal reorganization pathways which involved sliding or reptation of individual strands and 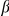-sheet subdomains relative to each other. All the observed oligomeric states therefore exhibit compact arrangements with a low radius of gyration [78]. These findings are consistent with a proposed aggregation mechanism that has been observed in simulations of various amyloidogenic peptides [35], [40], [43], [43,81] and has been also found experimentally [11], prevalent at high concentrations.

### Hydrophobic character of peptide and 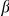-sheet content of aggregates are correlated

The assembly and ordering dynamics of the early oligomers are likely governed by the burial of hydrophobic side chains and intermolecular hydrogen bonding. The peptide self-assembly was found to be primarily accompanied by the desolvation of hydrophobic parts of the molecules. This is in line with the observations from various experimental and theoretical studies on low molecular weight oligomers [19], [28], [34], [40], [45], [46], [78], [82]–[84].

From both investigated peptide sequences, IB12 is the more hydrophobic, featuring a patch of adjacent hydrophobic residues. Interestingly, we found a decreased proportion of orientationally well-aligned IB12 peptide aggregates, while the amount of 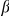-sheet structure was increased with respect to PHF6 aggregates of all observed sizes [34], [85]. At the same time we observed that the mutual association of smaller aggregates (condensation) was the preferred growth pathway for larger IB12 oligomers.

Here, an elevated level of backbone hydrogen bonding is directly correlated with a marked increase in hydrophobic burial, as well as the onset and magnitude of single 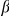-sheet formation and 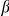-sheet stacking. The critical importance of nonpolar surface burial in peptide self-assembly has been specifically attributed to single 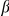-sheet layer stability and the lateral lamination of 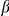-sheet [14], [39], [84], [86], [87].

### Kinetics impact the primary steps of amyloidogenic peptide assembly

We probed the structural characteristics of the spontaneously assembled aggregates and observed a rich variety of intermediates. A striking observation was the preferential anti-parallel inter-strand orientation in both the PHF6 and IB12 dimers, which could be directly traced back to key residue interactions in commonly observed encounter complexes, respectively. It is suggested that basic features like the 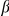-strand alignment (parallel vs. anti-parallel) can be kinetically determined at the early stages of assembly for aggregates as small as dimers [44].

In Figure S8, we show the projections of the spontaneously formed dimers together with the isolated dimers, which were additionally simulated on long time scales. The conformational distributions are comparable for the different PHF6 ensembles, whereas the IB12 ensembles differ substantially. The isolated IB12 dimers that were allowed to relax for 1 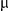
s, were mainly parallel in contrast to the spontaneously formed anti-parallel ones. We conclude that kinetic trapping was in particular relevant for the described anti-parallel IB12 dimer formation, which was in turn mainly driven by a hydrophobic effect. As seen from the transition networks and in the isolated dimer simulations, the two alignment states can inter-convert in the dimer nevertheless, as the barriers separating these states are rather small. For larger aggregates this interconversion is limited to edge strands. Hence, the non-equilibrium situation in our present study did not allow for structural relaxation of the spontaneously formed aggregates. The rapid oligomer growth therefore is the main factor for the observed kinetic control of dimer interfaces.

Preformed 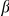-sheet dimers were found to be involved in the addition of isolated monomeric peptides in solution, as well as in the growth of larger aggregates. Do therefore kinetically trapped small aggregates determine the structural evolution of larger oligomers, as the presence of a number of alternative end-structures in the decamers suggest?

To answer this question we investigated if the preferentially anti-parallel strand alignment was also observed in the higher order oligomers due to kinetic trapping. We compared various dimer ensembles and show the projections of combined dimers obtained by decomposing trimer, tetramer and decamer aggregates into dimers (see Fig. S9). The decomposed multimeric PHF6 aggregates display a similar landscape to the spontaneously formed ‘true’ dimers. In the case of IB12, a mixed strand alignment pattern for trimers, tetramers and decamers is observed, which is different from the mainly anti-parallel ‘true’ dimers, but also not the same as found for isolated, relaxed dimers.

In fact, our results support the idea that a structure can be selected kinetically during early stages of assembly, where the nucleation barrier and hence production rate determines the abundance of the different aggregate morphologies and structural forms [37], [48]. This suggests that a rich structural heterogeneity or polymorphism on the dimer level can translate to the ensemble of oligomer conformations [40], [79], even at concentrations far below to the one investigated here [44]. Experimental evidence shows that alternative states of peptides in the condensed phase encompass a broad and diverse spectrum of oligomers and protofibrils, which themselves are polymorphic [15], [83]. Additionally, polymorphic forms of mature amyloid fibrils are well documented and can originate from variations in filament architecture and organization [6], [12], [17].

The study of short amyloidogenic peptides supports the notion that alternative packing schemes of highly ordered steric zipper conformations in the crystal structures and fibrils serve as a basis for molecular polymorphism [39], [58]–[61], [83]. We found a different strand alignment pattern in our simulations of spontaneously assembled PHF6 and IB12 oligomers than experimentally determined in the x-ray structures by Nelson and co-workers [58], although the peptide concentration and pH are in accord with the crystallization conditions. We assume that the protonation state of the C-terminus primarily affects the direction of strand alignment in the short steric zipper peptides. Since we considered only static protonation states throughout the simulations we expect a strong influence on PHF6 and IB12 peptide assembly. As a result, the aggregate conformations might be strongly affected by a dominant electrostatic effect of the termini. That is because the anti-parallel/in-register state was associated with the strongest attraction between the charged terminal residues. It is suggested that the packing of these peptides in the crystal is determined by a delicate balance of different factors [39], [40] (e.g. electrostatic contacts between symmetry mates, presence of solvent and counter ions), which implies alternative or co-existing 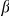-sheet bilayer conformers of similar stability. Furthermore, possible hierarchical or sequential assembly scenarios [39], [61], [88] can also affect the steric zipper structure selection and therefore render an extrapolation from the early oligomers in our simulations to the crystalline or fibrillar end-product challenging.

It is important to note that our present findings, however, do not rule out that the spine architecture of VQIVYK and VEALYL fibrils may resemble the steric zipper motifs identified by x-ray microcrystallography [58]. The spontaneously formed oligomers sample essential structural features of the steric zipper conformation: stacked 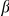-sheets with a dry interface, displaying tight side chain interdigitation. It is therefore conceivable that the observed the structural transitions at the decamer level are indicative of on-pathway sampling to the mature fibrils.

### Spontaneous oligomer formation occurs fast

The various spontaneously formed multimeric aggregates demonstrate that, the initially monomeric peptide molecules interact without encountering any major barriers. Furthermore, we saw that stable PHF6 and IB12 oligomer formation by association of smaller intermediates and remaining free monomers progressed rapidly, leading in all cases to one big aggregate. This indicates a fast oligomer growth up to a size of 10 peptide chains. This suggests that the primary aggregation and pre-nucleation stage is primarily an energetically downhill process. A recent Monte Carlo study on AcPHF6 oligomerization reports a similar scenario [36]. There the nucleation of a fibril competent species for further growth was not required until aggregates became larger than at least decamers.

The early and dynamic oligomers of minimal steric zipper peptides observed in our study were found to be pre-structured and did not condense in a fluctuating micelle-like arrangement, which are held together mainly by weak dispersion interactions as proposed for assemblies of longer peptides [19], [26], [89]. The conformational reorganizations necessary to access the highly ordered fibrillar state with a sterically complementary 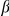-sheet interface mainly concern the peptide strand alignment and repacking of the side chains. The nucleation of a growth-competent steric zipper oligomer species might therefore be strongly disfavored by entropic arguments, thereby explaining the gap between the fast oligomerization as observed here and the known slow kinetics of in vitro fibril formation.

In this work we have concentrated on the primary aggregation events. The oligomeric end-states of our simulations show interesting structural reorganization dynamics that warrants further analysis. This will be the subject of a following study.

### Conclusion

We have reported on atomistic MD simulations of the unbiased spontaneous aggregation process of PHF6 and IB12 steric zipper peptides from unstructured monomers to 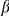-sheet rich oligomeric assemblies. The current study and detailed analyses of eight independent simulations for a total combined time of 2.4 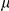
s per peptide system highlights several findings that in particular address the course of primary events in peptide assembly. First, a rapid formation of a heterogeneous ensemble of 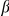-sheet rich oligomer structures was observed, where kinetically trapped aggregate intermediates affected the structural evolution of larger assemblies. Second, oligomerization was found to proceed via a combination of polymerization and condensation mechanisms. Finally, we reveal that the observed diverse association and reorganization dynamics are governed by the characteristics of peptide sequence and oligomer size.

A thorough characterization of the heterogeneity in molecular dynamics and structures of the low molecular weight oligomers may hold the key to understand the profound differences in macroscopic fibril growth kinetics [26], [88], [90], [91] and the observed rich structural diversity of aggregate states [17], [59]–[61], [83], [89]. Specifically, one may speculate that the structural relations between crystalline and fibrillar polymorphs of amyloidgenic peptides may be only resolved by tracing and determining the characteristics of the oligomeric conformational states from which either of the species originate.

## Methods

### Simulation Setup and Procedure

#### Simulated Systems

An overview of the simulated peptide systems is given in Table 1. The simulations were categorized according to the name of the peptide and the starting configuration and have a length of 4.8 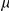
s altogether.

#### Initial Conformations

Conformational ensembles containing 1000 peptide structures each were generated with CONCOORD [92] based on the atomic coordinates of the PDB crystal structures: 2ON9 (VQIVYK; PHF6) and 2OMQ (VEALYL; IB12) [58], respectively. Only topological constraints were defined, resulting in random starting configurations for the simulations.

The individual simulations were set up according to the following protocol: 10 different peptide conformations were randomly chosen from the pre-generated structure ensemble and placed randomly in position and orientation in a cubic box (1000 nm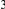) to result in a concentration of 16.6 mM. This procedure was applied to reduce the bias from the individual peptide's starting position and configuration and to ensure a fully monomeric starting configuration for each of the conducted simulation runs, respectively.

Subsequently all systems were solvated with explicit water molecules. The protonation state of the peptides was at pH 7 for all simulations of the PHF6 peptides according to the one in solution. For the IB12 peptide simulations and consistent with experimental conditions at pH 2.5 [54], [58], the C-terminus and the glutamate side chains were assumed to be protonated. Counter-ions (Na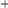, Cl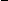) were added to yield an appropriate ionic strengh (0.15 M) and to neutralize the net system charge. The simulation systems were each comprised of roughly 100×10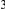 atoms. A typical simulation box is shown in Figure S9.

After the system preparation an energy minimization using steepest descent was performed.

### MD Setup

All MD simulations were carried out using the GROMACS software package (version 4.0) [93]–[95]. The Berendsen coupling algorithm [96] was applied to keep the pressure constant by coupling the system to a pressure bath of 1 bar (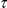 = 1 ps). Velocity rescale [97] was applied for temperature coupling to a temperature bath of 310 K. Initial velocities were taken from a Maxwellian distribution at 310 K.

All protein bonds were constrained with the P-Lincs algorithm [98]. Virtual interaction sites of all hydrogen atoms were introduced, thereby removing all internal vibrational degrees of freedom. This allowed us to use an integration time step of 5 fs while maintaining energy conservation [93], [99]. Neighbor lists for non-bonded interactions were updated every 5 steps.

For production runs the GROMOS96 43A1 [100], [101] force field and the SPC water model [102] were used. Water molecules were constrained using SETTLE [103]. The short-ranged non-bonded interactions, namely van der Waals and electrostatic were cut-off at 1.4 nm and 0.9 nm, respectively.

All simulations were carried out using periodic boundary conditions and the Particle Mesh Ewald (PME) [104], [105] method. The electrostatic interactions with PME were calculated at every step with a grid spacing of 0.12 nm. The relative tolerance at the cut-off was set at 10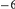, electrostatic interactions for a distance smaller than the real space cut-off were calculated explicitly.

### Analysis

Samples for analysis were taken every 2.5 ps from the collected trajectories.

#### Definitions of general and 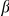-sheet aggregates

In order to quantitatively probe the association state of the peptides at any given time we pursued a hierarchical classification of the formed aggregates and their numerous conformations:

- Pairwise inter-peptide contact analysis was used to identify the individual aggregates, defined as general peptide assemblies: Peptides which shared an inter-chain residue contact were assumed to be within the same aggregate. For any two peptides 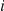, 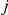 an inter-chain contact was considered to be formed if any heavy atom of peptide 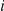 was within a distance of 0.45 nm from any heavy atom of peptide 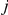.
- To trace not only the general peptide association but also any sort of transition to ordered species we defined assemblies of peptides aggregated into intermolecular 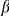-sheets: Any two peptides which shared two consecutive inter-chain 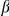-sheet contacts as defined by the DSSP algorithm [75] were considered to constitute a 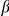-sheet aggregate, a subpopulation of the above considered general peptide aggregates.

#### Orientational order parameter P2

The nematic order parameter (P2) of the system yields information about the extent of alignment and relative orientation of the individual peptides. We defined a suitable molecular vector (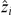), here the unit vector linking the C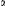-atoms of the second to fifth residue of the 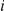-th peptide.
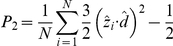
The order parameter P2 was calculated using the WORDOM program package [106].

#### Oligomer decomposition and dimer relabeling

The pathways of PHF6 and IB12 peptide aggregation were mapped and analyzed through the perspective of a minimal oligomer, i.e., the peptide dimer. All aggregates consisting of three, four or ten peptides were separately decomposed into smaller dimeric subsystems based on a minimal distance criterion. Afterwards, these decomposed structures were consistently relabeled, clustered and transition pathways between clusters constructed:

The decomposition of higher oligomers into dimeric structures was performed separately for trajectories which contained all trimeric, tetrameric and decameric aggregates observed in all simulations for both, PHF6 and IB12, peptides. For each trajectory frame a neighbor list of peptides was generated for each molecule. Peptide 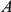 was added to the neighbor list of peptide 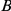 if at least one heavy atom of 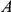 and 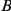 were less than 0.45 nm apart. That way all possible combinations of dimers for each molecule and the molecules in its neighbor list were saved. Only unique dimer combinations were considered by removing identical and permuted combinations, e.g. dimers 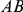 and 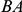 were treated as equivalent and hence one of them discarded. Thereby a conformational ensemble of dimeric structures was obtained, constructed from a specific oligomeric state (trimer, tetramer and decamer).

Both, the clustering of the dimeric structures and mapping them onto principal components, requires superposition to a reference structure. In order to fit structures consistently, peptides forming dimers were labeled such that similar molecules were given the same chain identifier (ID).

In the first step of this relabeling procedure, peptides in a dimeric structure were assigned with individual IDs and fit to a reference structure. In the second step, IDs for the peptides were interchanged and fitting was performed again. Labels that resulted in a lower root mean square deviation (RMSD) between the dimer of interest and the reference structure were kept. Main-chain and C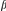 atoms were used for fitting and the RMSD calculation. To prevent the dependence of labeling on the reference structure, an iterative relabeling scheme was applied. Initially, relabeling was performed using a peptide dimer built from the respective crystal structure conformation as a reference. The average structure of the relabeled trajectory was calculated. The relabeling procedure was repeated using this average structure as a reference. The cycle was repeated until all structures in the trajectory were labeled identically in two subsequent iterations.

#### PCA and k-means clustering

Principal component analysis (PCA) [107] was carried out over the conformations of the spontaneously formed PHF6 and IB12 dimers, respectively. The covariance matrix of atomic displacement was constructed and diagonalized for the coordinates of main-chain and C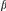 atoms. All structures were superimposed to the respective average structure calculated over all the dimer conformations prior to analysis. The conformational ensemble of spontaneously formed PHF6 and IB12 dimers were projected onto the first three eigenvectors to obtain a mapping into a space of reduced dimensionality, respectively.

For the conformational clustering of dimeric structures, coordinates of the main-chain and C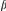 atoms were extracted from each peptide dimer. RMSD values between dimeric structures were used as a distance measure for k-means clustering. The implementation of the k-means Hartigan-Wong algorithm [108] in the statistical software package R [109] was used for clustering. Cluster centers were selected according to the global k-means algorithm [110], the optimal number of clusters was determined by the Krzhanowski-Lai criterion [111]. Structures with the lowest RMSD value to the geometrical centers of the clusters were selected as representatives. The selection of cluster centers and numbers was performed only for the spontaneously formed dimer conformations of PHF6 and IB12, respectively. The decomposed trajectories of trimers and tetramers were clustered using these respective cluster centers and numbers.

#### Pathway mapping

Transition networks were constructed based on the conformational clustering. Nodes represent clusters and edges the transitions between them. The size of a node was determined by counting occurrences of a structure in a certain cluster. Transitions between two clusters were counted if dimer conformations traversed to another cluster in the subsequent time step (frame). If a dimer conformation disassembled or formed a larger aggregate for at least one time step, a transition out of the node (cluster) was counted. If a dimer conformation was formed, a transition into the node (cluster) was counted. Pathway network construction was performed with Cytoscape [112]. Node and edge sizes were normalized for each network separately.

### Visualization

Molecular images in the main text and PCA projections were rendered using VMD [113] and Tachyon [114]. The pathway networks were visualized using the Cytoscape package [112].

## Supporting Information

Figure S1 **Typical starting configuration: A cubic simulation box with 10 monomeric peptides, ions and explicit water molecules.** The peptide backbones are depicted in cartoon representation and side chain atoms as sticks, sodium and chloride ions as spheres and water molecules as transparent sticks.
(TIFF)

Figure S2
**Time evolution of aggregate and** 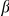 **-sheet sizes for all 8 independent PHF6 simulations.**
(TIFF)

Figure S3
**Time evolution of aggregate and** 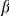 **-sheet sizes for all 8 independent IB12 simulations.**
(TIFF)

Figure S4
**Oligomer formation and growth proceeded by bimolecular associations.** The scheme highlights all possible bimolecular reactions up to the decamer (non-white boxes) and their respective number of occurrence in all of the PHF6 (A) and IB12 (B) simulations. The values in the boxes indicate the total number of observed bimolecular association and dissociation reactions between aggregates of size 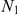 and 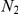.(TIFF)

Figure S5 **Analysis of hydrophobic solvent accessible surface area (hSAS) for all 8 independent PHF6 (A) and IB12 (B) simulations, respectively.**
(TIFF)

Figure S6 **Residue-Residue contact map for individual PHF6 dimer clusters.** The calculation was performed separately for the dimer structures of each of the six identified conformational states shown in Figure 7. The map is colored by the average occurrence of inter-peptide residue pairs, which share at least one heavy atom contact. The scale is given on the right top.
(TIFF)

Figure S7 **Residue-Residue contact map for individual IB12 dimer clusters.** The calculation was performed separately for the dimer structures of each of the five identified conformational states shown in Figure 8. The map is colored by the average occurrence of inter-peptide residue pairs, which share at least one heavy atom contact. The scale is given on the right top.
(TIFF)

Figure S8
**Ensemble of spontaneously formed and isolated PHF6 and IB12 peptide dimers.** Projections of various dimer ensembles as a function of radius of gyration (R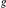) and the C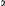 root-mean-square deviation (RMSD) to the known crystal structure conformation of the PHF6 and IB12 peptides (2ON9, 2OMQ) are depicted, respectively. Spontaneously formed dimers (A - PHF6 and C - IB12) and isolated dimer conformations, which were simulated additionally on long time scales (B - PHF6 and C - IB12) are shown. The projections of the isolated dimer conformations were obtained from two 1 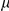
s long simulations, respectively. The normalized frequency of occurence scale is given on the right.(TIFF)

Figure S9
**Ensemble of PHF6 and IB12 peptide dimers derived from different aggregation states.** Projections of various dimer ensembles as a function of radius of gyration (R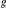) and the C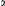 root-mean-square deviation (RMSD) to the known crystal structure conformation of the PHF6 and IB12 peptides (2ON9, 2OMQ) are depicted, respectively. Spontaneously formed dimers (A - PHF6 and E - IB12) and ensemble of trimers, tetramers and decamers (B, C, D - PHF6 and F, G, H - IB12) are shown, respectively. Ensembles of higher order oligomers were obtained by decomposition of the respective multimer into dimers (see Methods). The normalized frequency of occurence scale is given on the right.(TIFF)

Movie S1 **Molecular dynamics simulation of spontaneous VQIVYK peptide aggregation.**
(MPG)

Text S1 **Evaluation and validation: a consensus force field approach.**
(PDF)
