# Supplementary figures and images for: Mapping the Conformational Dynamics and Pathways of Spontaneous Steric Zipper Peptide Oligomerization
Source: PLoS One. 2011 May 3;6(5):e19129. doi: 10.1371/journal.pone.0019129 (PMC3086902; doi:10.1371/journal.pone.0019129)

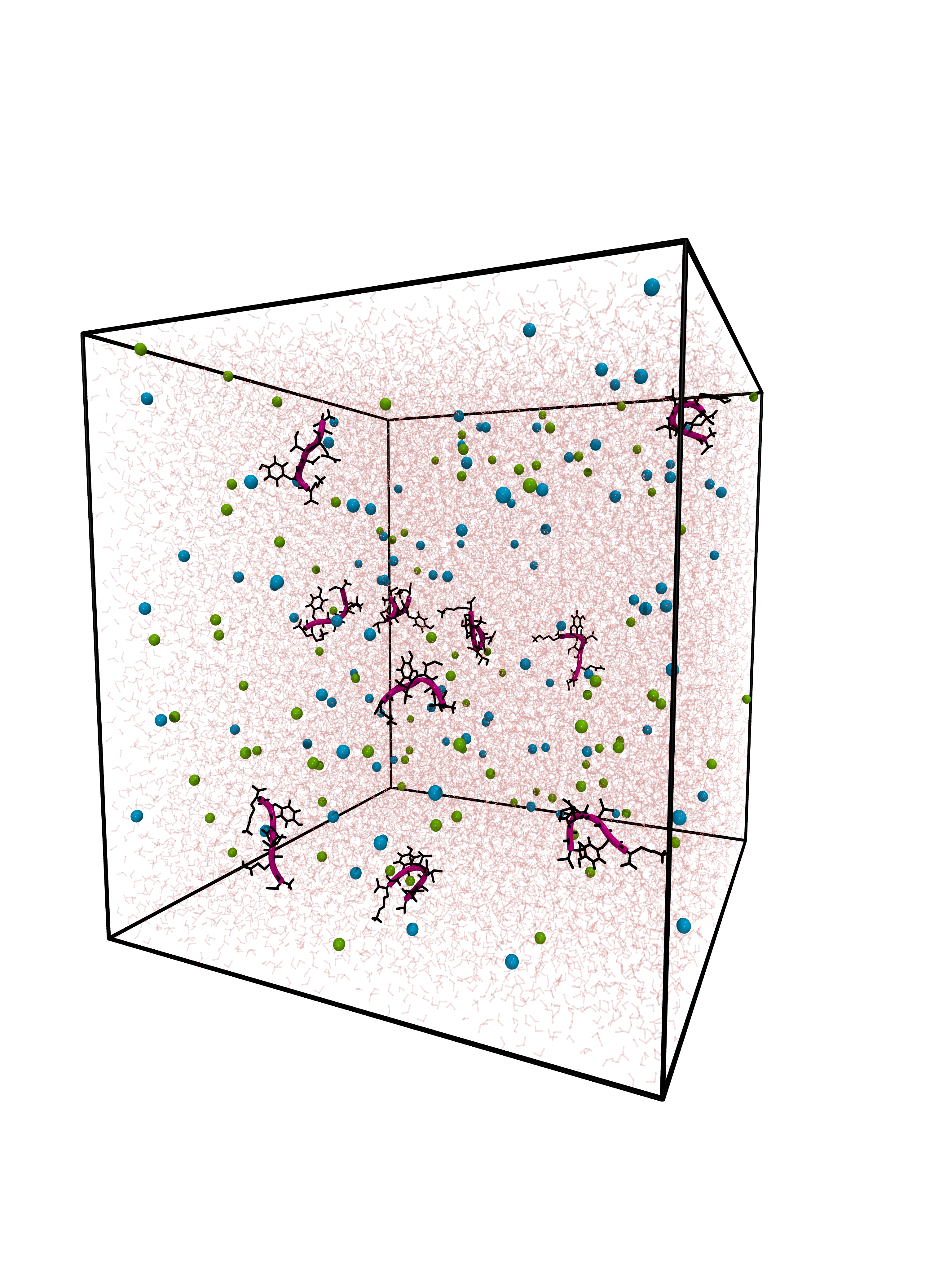

Supplement: Figure S1 — Typical starting configuration: A cubic simulation box with 10 monomeric peptides, ions and explicit water molecules. The peptide backbones are depicted in cartoon representation and side chain atoms as sticks, sodium and chloride ions as spheres and water molecules as transparent sticks. (TIFF) [file pone.0019129.s001.tiff]

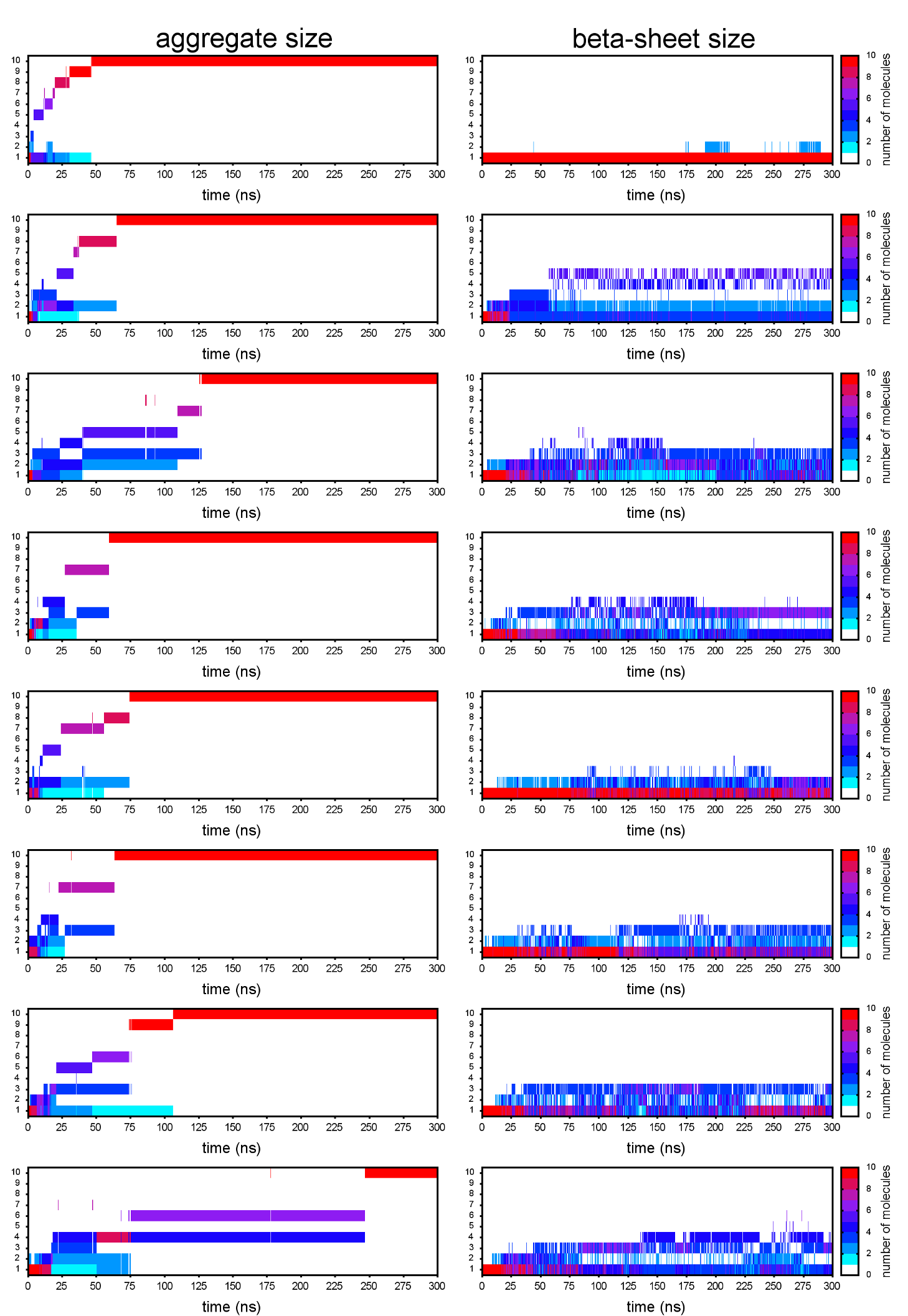

Supplement: Figure S2 — Time evolution of aggregate and -sheet sizes for all 8 independent PHF6 simulations. (TIFF) [file pone.0019129.s002.tiff]

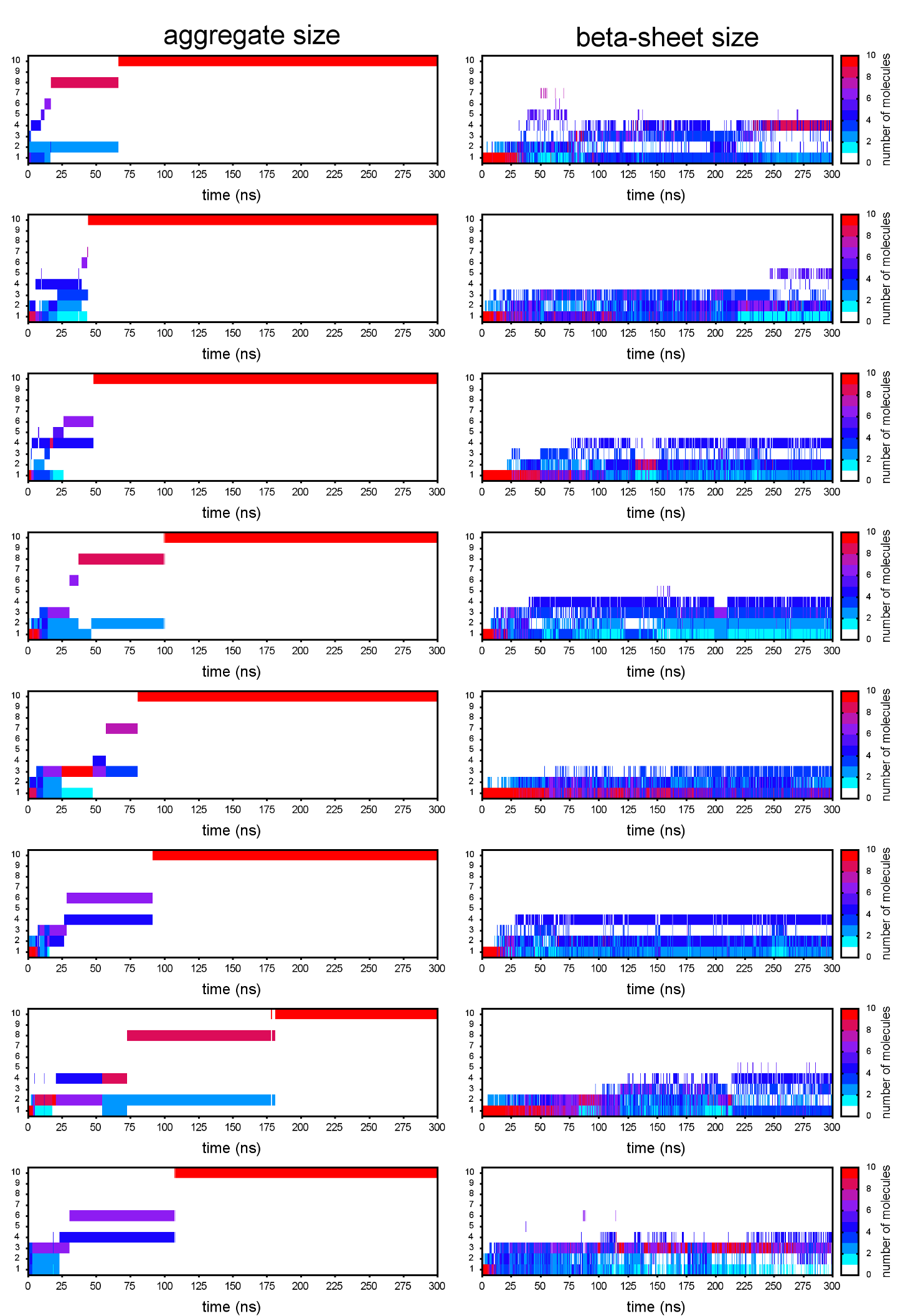

Supplement: Figure S3 — Time evolution of aggregate and -sheet sizes for all 8 independent IB12 simulations. (TIFF) [file pone.0019129.s003.tiff]

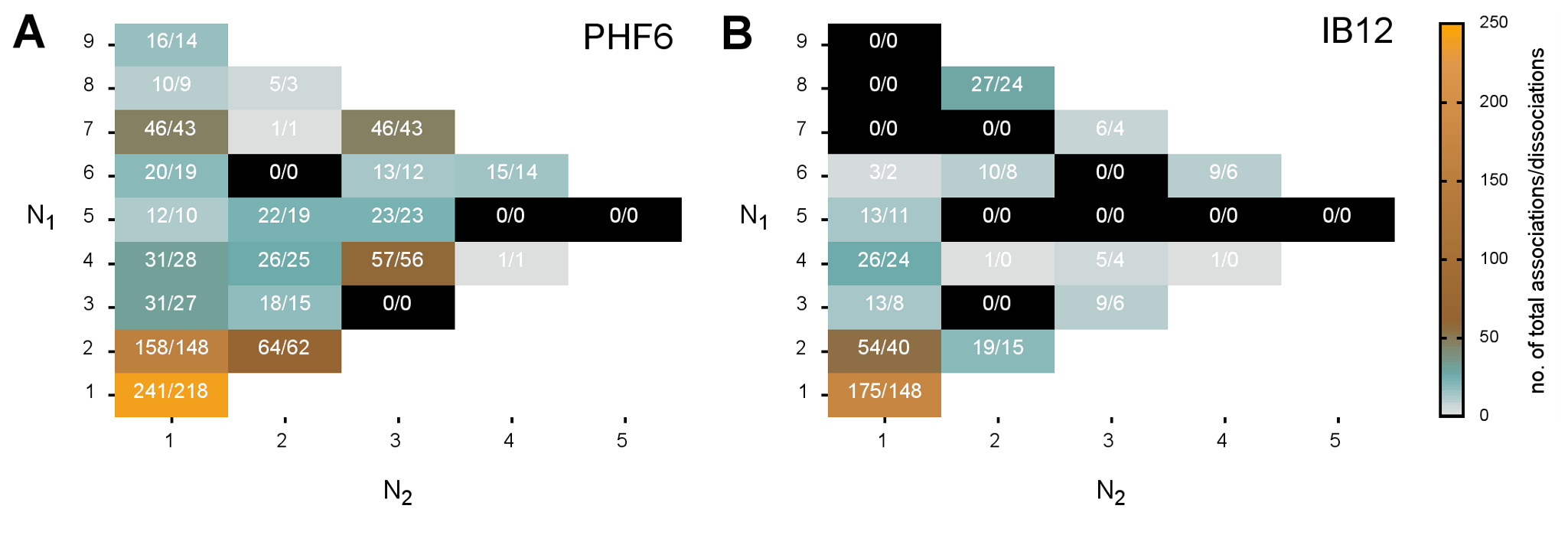

Supplement: Figure S4 — Oligomer formation and growth proceeded by bimolecular associations. The scheme highlights all possible bimolecular reactions up to the decamer (non-white boxes) and their respective number of occurrence in all of the PHF6 (A) and IB12 (B) simulations. The values in the boxes indicate the total number of observed bimolecular association and dissociation reactions between aggregates of size and . (TIFF) [file pone.0019129.s004.tiff]

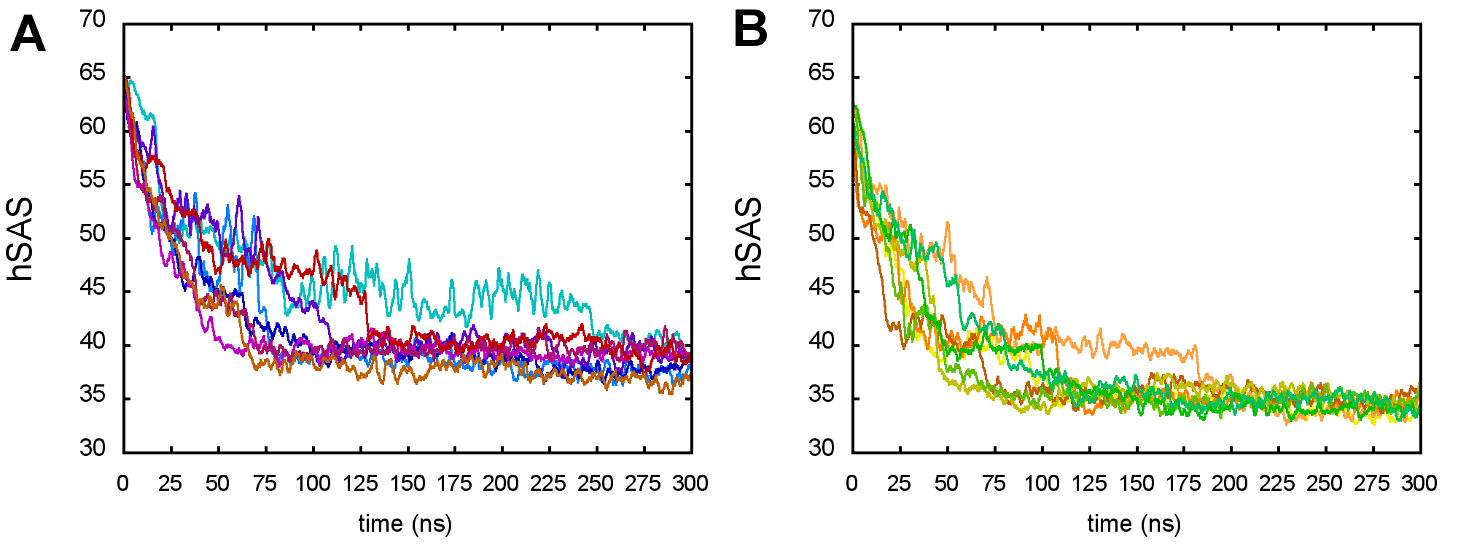

Supplement: Figure S5 — Analysis of hydrophobic solvent accessible surface area (hSAS) for all 8 independent PHF6 (A) and IB12 (B) simulations, respectively. (TIFF) [file pone.0019129.s005.tiff]

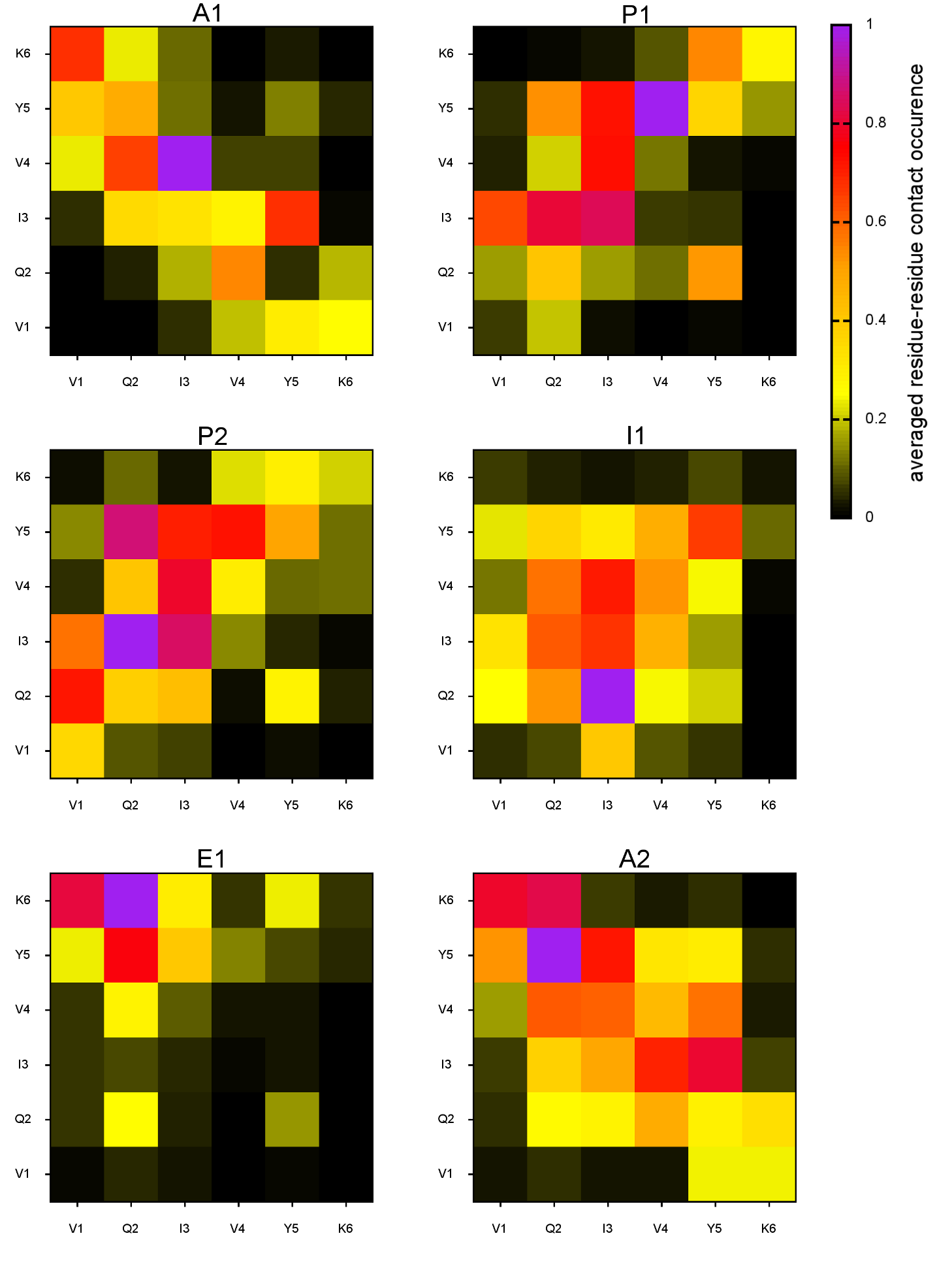

Supplement: Figure S6 — Residue-Residue contact map for individual PHF6 dimer clusters. The calculation was performed separately for the dimer structures of each of the six identified conformational states shown in Figure 7. The map is colored by the average occurrence of inter-peptide residue pairs, which share at least one heavy atom contact. The scale is given on the right top. (TIFF) [file pone.0019129.s006.tiff]

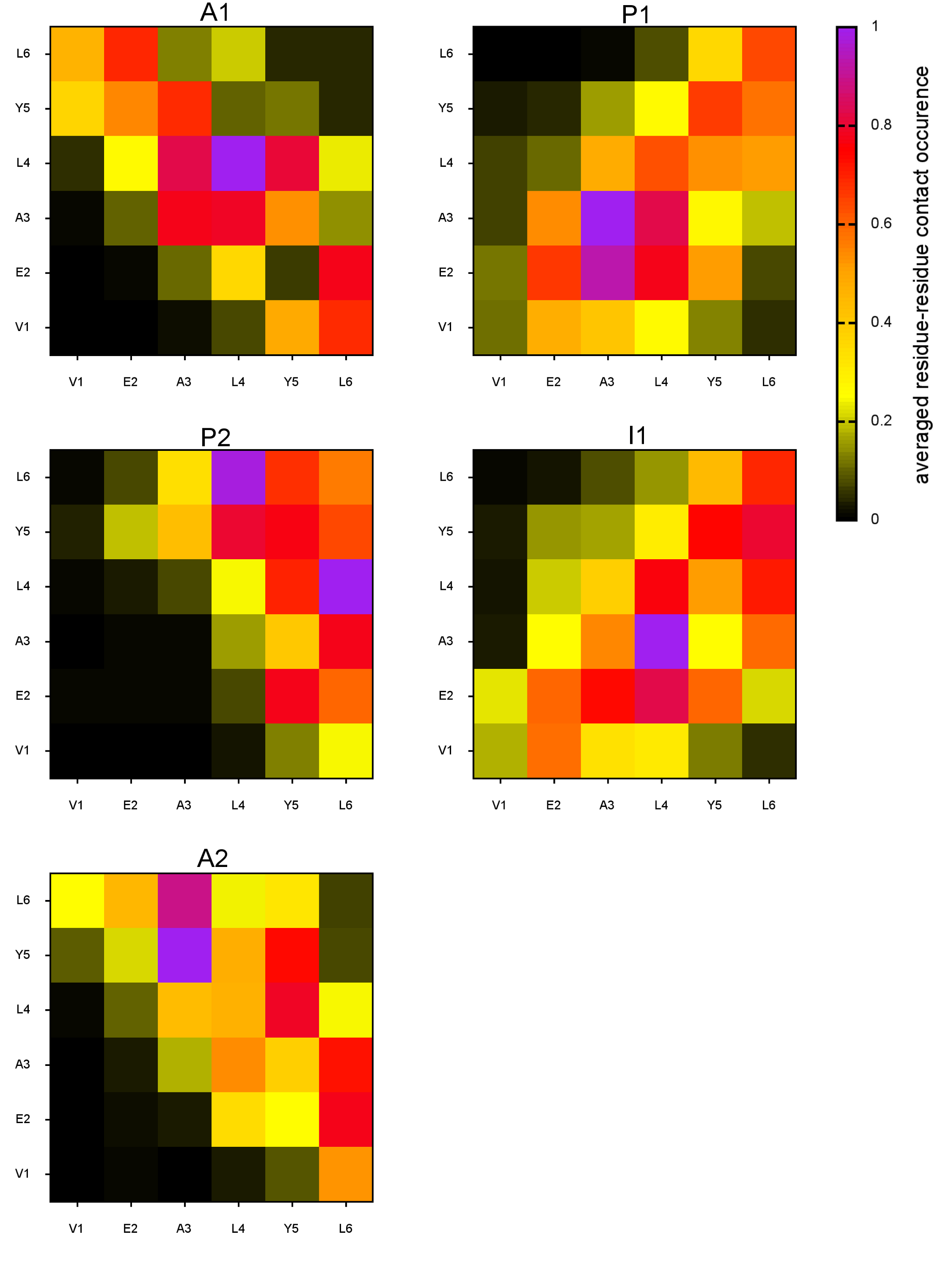

Supplement: Figure S7 — Residue-Residue contact map for individual IB12 dimer clusters. The calculation was performed separately for the dimer structures of each of the five identified conformational states shown in Figure 8. The map is colored by the average occurrence of inter-peptide residue pairs, which share at least one heavy atom contact. The scale is given on the right top. (TIFF) [file pone.0019129.s007.tiff]

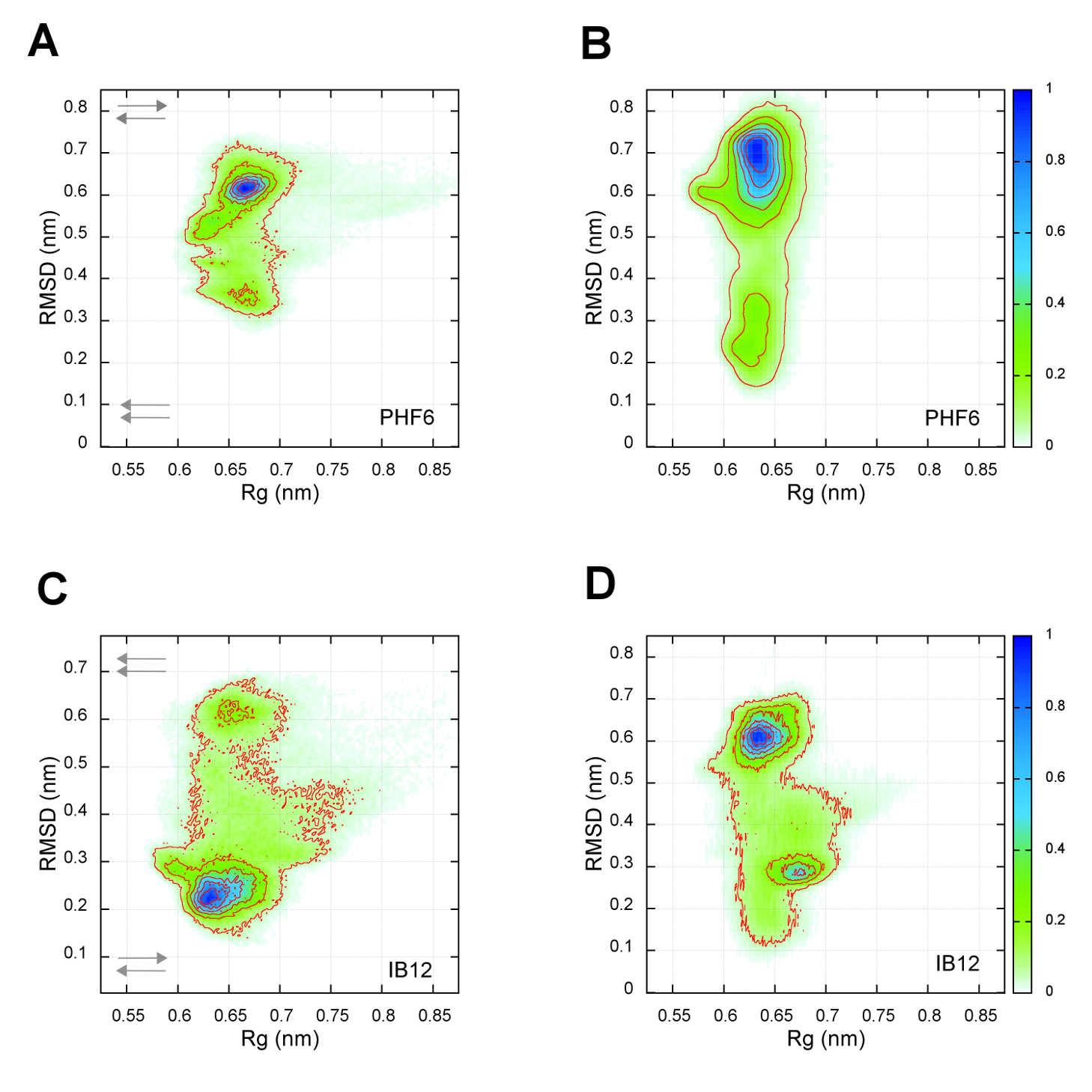

Supplement: Figure S8 — Ensemble of spontaneously formed and isolated PHF6 and IB12 peptide dimers. Projections of various dimer ensembles as a function of radius of gyration (R) and the C root-mean-square deviation (RMSD) to the known crystal structure conformation of the PHF6 and IB12 peptides (2ON9, 2OMQ) are depicted, respectively. Spontaneously formed dimers (A - PHF6 and C - IB12) and isolated dimer conformations, which were simulated additionally on long time scales (B - PHF6 and C - IB12) are shown. The projections of the isolated dimer conformations were obtained from two 1 s long simulations, respectively. The normalized frequency of occurence scale is given on the right. (TIFF) [file pone.0019129.s008.tiff]

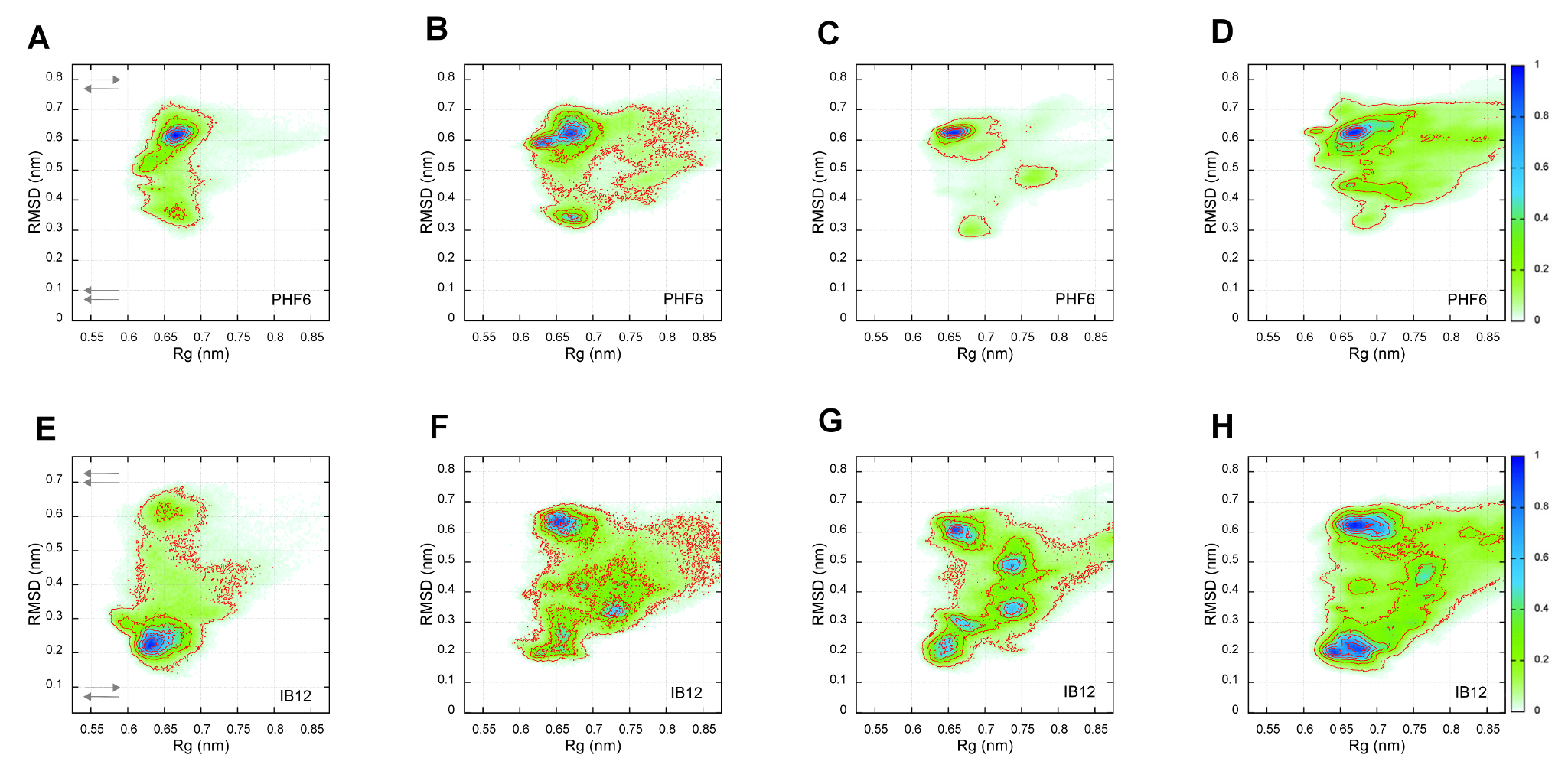

Supplement: Figure S9 — Ensemble of PHF6 and IB12 peptide dimers derived from different aggregation states. Projections of various dimer ensembles as a function of radius of gyration (R) and the C root-mean-square deviation (RMSD) to the known crystal structure conformation of the PHF6 and IB12 peptides (2ON9, 2OMQ) are depicted, respectively. Spontaneously formed dimers (A - PHF6 and E - IB12) and ensemble of trimers, tetramers and decamers (B, C, D - PHF6 and F, G, H - IB12) are shown, respectively. Ensembles of higher order oligomers were obtained by decomposition of the respective multimer into dimers (see Methods). The normalized frequency of occurence scale is given on the right. (TIFF) [file pone.0019129.s009.tiff]
