# Supplementary material for: Mapping the Conformational Dynamics and Pathways of Spontaneous Steric Zipper Peptide Oligomerization
Source: PLoS One. 2011 May 3;6(5):e19129. doi: 10.1371/journal.pone.0019129 (PMC3086902; doi:10.1371/journal.pone.0019129)
Supplement: Text S1 — Evaluation and validation: a consensus force field approach. (PDF) [file pone.0019129.s011.pdf]

Supporting Information

Dirk Matthes, Vytautas Gapsys and Bert L. de Groot

Text S1

Evaluation and validation: a consensus force field approach

To evaluate and validate our findings for the simulations of spontaneous peptide aggregation with GROMOS96 43A1, a consensus force field approach for biomolecular aggregation was carried out. Various molecular mechanics force fields were compared in their description of selected peptide dimer conformations. The additional force field variants included in this study were AMBER99SB and CHARMM27. We chose representative dimeric states as the initial conformations (see Figure 1 and 2) for both peptide sequences (PFH6, IB12): Preformed β-sheets (*pre*) in an ordered, either parallel or anti-parallel orientation. The preformed dimers were included as a reference state to probe the stability of a fibril-like peptide arrangement. Additionally we investigated peptide dimers which resemble typical first encounter complexes (*enc*) or transition states, where only a few atomic contacts or interactions via hydrogen bonds are present. These dimer structures were extracted from the reported ensembles obtained from spontaneous aggregation simulations in the GROMOS96 43A1 force field. A summary of all trajectories carried out and their corresponding starting structure is presented in Table 1.

Table 1. Summary of performed validation simulations.

**Starting configuration**	**number of runs per force field**	**sim. length (ns)**
PHF6-pre1 (parallel, in-register)	10	15
PHF6-pre2 (anti-parallel, off-register)	10	15
PHF6-enc1 (collapsed)	10	15
PHF6-enc2 (docked)	10	15
IB12-*pre1* (anti-parallel, in-register)	10	15
IB12-*pre2* (parallel, in-register)	10	15
IB12-*enc1* (orthogonal, beta-bridge)	10	15
IB12-*enc2* (docked)	10	15

MD Setup

All additional MD simulations were carried out using the GROMACS software package (version 4.0) (1–4).

The results obtained from the GROMOS96 43A1 trajectories were validated against simulations with the CHARMM27 force field (5) (with backbone potential [CMAP] correction and TIP3P water model) and the AMBER99SB force field (6) (with TIP3P water model) using the GROMACS ports by Bjelkmar and Sorin, respectively (7, 8). The parameters to model the protonated C-terminal leucine in the AMBER99SB force field were taken by analogy from Best and Hummer (9). Ion parameters from Joung et al. (10) were used for the simulations with the AMBER force field. The following simulation input parameter were used: For the CHARMM27 force field van der Waals interactions were switched off between 1.0 to 1.2 nm and short-range electrostatic interactions were cut off at 1.2 nm. Short-range cutoffs of 1.0 nm, and 0.9 nm were used for van der Waals and electrostatic interactions, respectively when employing the AMBER99SB force field. In each case simulations were run using a 4 fs time step. The simulation protocol for the GROMOS96 43A1 was the same as reported in the Methods section of the article. All simulations were carried out using periodic boundary conditions and the Particle Mesh Ewald (PME) (11, 12) method. The electrostatic interactions with PME were calculated at every step with a grid spacing of 0.12 nm. The relative tolerance at the cut-off was set at 10^{-6}, electrostatic interactions for a distance smaller than the real space cut-off were calculated explicitly.

The different peptide dimer systems were prepared in a cubic box (200 nm^3), respectively. The structures were subsequently solvated in explicit water molecules and ions were added according to the protocol outlined in the Methods section of the article. The system size for this setup was around 20000 atoms, respectively. We used the dimeric crystal structure conformation (PDB: 2OMQ - IB12) and (PDB: 2ON9 - PHF6), as well as two partially aggregated dimer structures which were extracted from the spontaneous simulations.

Comparison of sampled conformations

A comparison of the sampled conformations in the different force field by means of radius of gyration and the C_α RMSD of the structures is shown in Figure 1 and 2. We found a similar behavior and stability for the preformed β-sheet complexes (pre1, pre2) of PFH6, as well as IB12 in all tested force fields within the probed 15 ns timescale in all the validation simulations. An analysis of the conformations sampled starting from the initially docked, encounter complexes however show clear differences. For all GROMOS96 43A1 simulations compact structures are found, reflected in the distribution of conformations around low values of the radius of gyration. The encounter complexes do yield parallel and anti-parallel dimers with β-sheet content. A notable feature in the projection of R_g and C_α RMSD for both peptide sequences is the sampling of a region around (R_g: 0.65, RMSD: 0.45) with the GROMOS43A1 force field. Corresponding structures are collapsed and bent peptide chains with a varying amount of interpeptide backbone hydrogen bonds. This region is not sampled in the AMBER99SB and CHARMM27 simulations. Furthermore, the peptide dimers starting from encounter conformations do not adopt ordered β-sheet dimers in AMBER99SB and CHARMM27, and have the propensity to dissociate. The encounter complex dimers differ in their kinetic stability in the different force fields, as indicated by the lifetime and number of transitions between associated and dissociated state. By counting the individual dissociation and association events within the first 15 ns of each trajectory, we found irreversible displacements of peptide chains in most of the AMBER99SB and CHARMM27 *enc* simulations, whereas there was almost no dimer dissociation found in GROMOS96 43A1 (see Table 2). The average time spent in the aggregated state was in all cases significantly higher for GROMOS96 43A1 simulations.

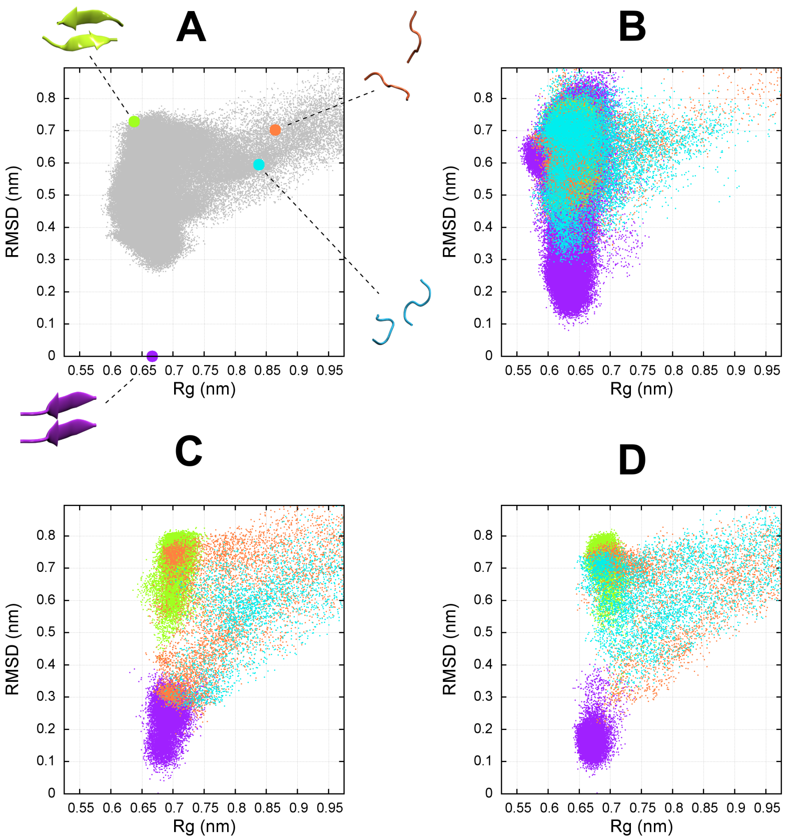

Figure 1. Projection of all sampled PHF6 dimer conformations as a function of the radius of gyration and the C_α RMSD to the parallel dimer reference structure for: spontaneous formed dimers as reported in the article (A), GROMOS96 43A1 (B), AMBER99SB (C), CHARMM27 (D). The initial conformations for *pre* and *enc* simulations are indicated by a purple (*pre1*), green (*pre1*) and blue (*enc1*) and orange (*enc2*) dot, respectively. The projections of the conformations obtained with the GROMOS96 43A1 force field include two extended *pre1* simulations (each 1 μs long) simulations as reported in the main article.

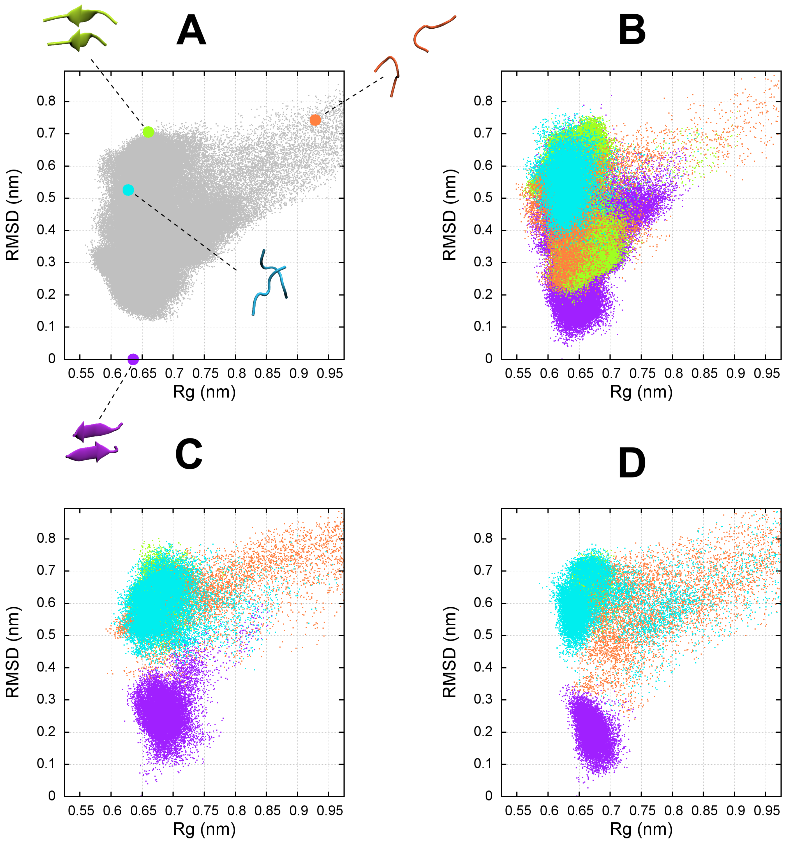

Figure 2. Projection of all sampled IB12 dimers as a function of the radius of gyration and the C_α RMSD to the anti-parallel dimer reference structure for: (A) spontaneous formed dimers (B) GROMOS96 43A1 (C) AMBER99SB (D) CHARMM27. The initial conformations for *pre* and *enc* simulations are indicated by a purple (*pre1*), green (*pre1*) and blue (*enc1*) and orange (*enc2*) dot, respectively. The projections of the conformations obtained with the GROMOS96 43A1 force field include one *pre1* and one *pre2* simulation (each 1 μs long) simulations as reported in the main article.

Table 2. Total number of peptide dimer complex dissociation (d) and association (a) events, as well as average fraction of time spend in aggregated state.

**Initial dimer configuration**	**GROMOS96 43A1** $\mathbf{n}_d/\mathbf{n}_a$ ($\mathbf{t}_{aggregated}$)	**AMBER99SB** $\mathbf{n}_d/\mathbf{n}_a$ ($\mathbf{t}_{aggregated}$)	**CHARMM27** $\mathbf{n}_d/\mathbf{n}_a$ ($\mathbf{t}_{aggregated}$)
PHF6-*pre1*	0/0 (1.00)	0/0 (1.00)	0/0 (1.00)
PHF6-*pre2*	0/0 (1.00)	0/0 (1.00)	0/0 (1.00)
PHF6-*enc1*	15/15 (0.77)	35/29 (0.44)	47/39 (0.30)
PHF6-*enc2*	2/1 (0.91)	55/48 (0.21)	45/34 (0.33)
IB12-*pre1*	0/0 (1.00)	0/0 (1.00)	0/0 (1.00)
IB12-*pre2*	0/0 (1.00)	0/0 (1.00)	0/0 (1.00)
IB12-*enc1*	0/0 (1.00)	3/3 (0.98)	16/16 (0.90)
IB12-*enc2*	8/6 (0.74)	55/48 (0.39)	57/55 (0.39)

Secondary structure propensities

The realistic preferential formation and representation of secondary structure is a critical prerequisite for the successful study of in silico peptide folding and aggregation (13). A DSSP analysis over all conformations sampled from 5 to 15 ns (discarding the first 5 ns) of each trajectory was carried out and averaged fractions of secondary structure elements obtained. We analyzed preformed and encounter complex dimers separately because we expect a bias from the initial starting structure on the simulated timescales reported here.

For the preformed dimers of PHF6 and IB12 we found β-sheet and random coil to be the dominant secondary structure elements. For the PHF6 peptide only half of the initial amount of β-sheets were retained and rather bent conformations were sampled (see Figure 3A and C). There was no apparent significant difference when comparing the results for the preformed dimers in the different force fields. While CHARMM27 and GROMOS96 43A1 give very similar results, the AMBER99SB force field shows in both cases a slight tendency towards less β-sheet and more coil structures. We observed fluctuating amount of β-sheet content for the preformed dimers in all force fields, but only a slight decrease over time after a fast initial relaxation phase.
For the encounter complex simulations, we found overall mainly coil and bend structures for both peptide sequences. Additionally, a non-negligible amount of turn conformations and isolated β-bridges was found for the IB12 peptide. Although there were no large differences among the different force fields evident, some trends could be seen. The GROMOS96 43A1 simulations sampled extended β-sheet and isolated β-bridge structures most frequently, as well as the lowest percentage of coil and no helical conformations. For both peptides sequence the CHARMM27 force field yielded the smallest amount of β-sheet, but high fractions of coiled conformations were present. The AMBER99SB force field performed overall comparable to the CHARMM27 force field, although the sampling of β-sheet and turn conformations was slightly higher.

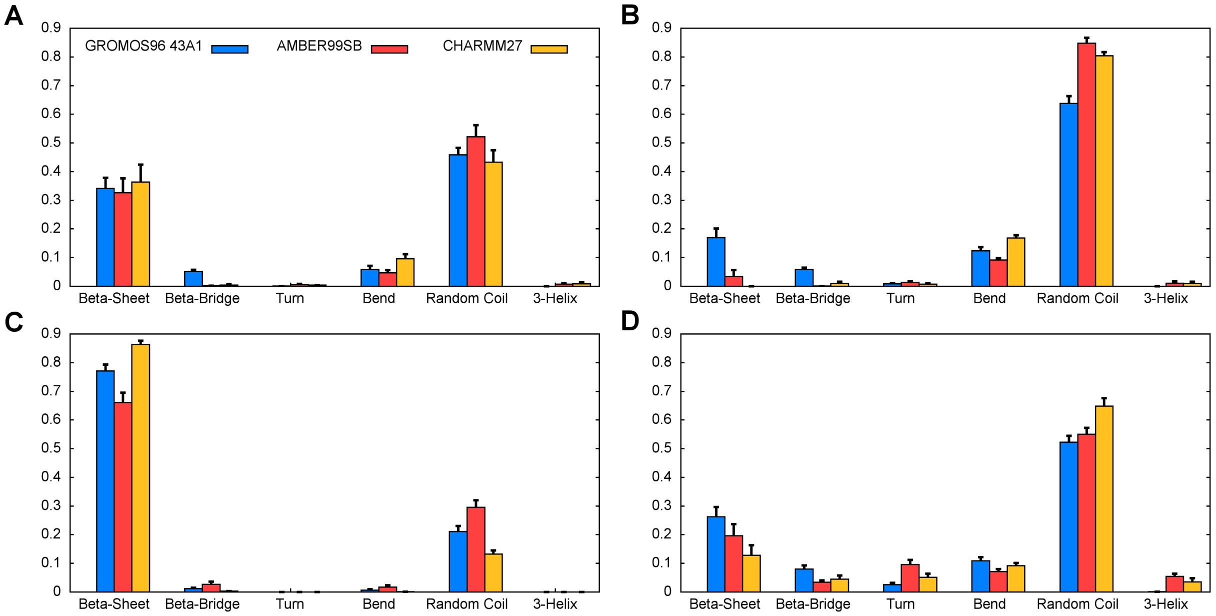

Figure 3. Averaged fractions of secondary structure elemtents found for the peptide dimers as obtained from DSSP analysis: PHF6-*pre1+2* (A), PHF6-*enc1+2* (B), IB12-pre1+2 (C), IB12-enc1+2 (D)

Summary

To elicit if our biomolecular aggregation study was sensitive to the choice of force field, we performed a set of validation experiments using representative dimeric states as starting structures for a number of replicate simulation runs. The results obtained here justify our conclusions made about spontaneous peptide oligomerization, but also point us to discuss important force field dependent differences: In terms of cross validation of MD force fields, the consensus approach presented here indicates that the different force field variants perform similar when preformed β-sheet rich dimers were examined, but differ in the description of transient encounter complexes. Preformed, ordered peptide dimers, including the crystalline conformation show similar structural characteristics and stability in all evaluated force fields. We made sure that the force fields selected for this study are not biased to particular secondary structures elements (13). And indeed we found minor differences in the propensity of secondary structure formation, when starting from preformed dimers. The description of the dimeric encounter complexes was found to differ. The peptide dimer preferentially adopt compact or collapsed conformations and showed the highest tendency to form extended β-sheets in the GROMOS96 43A1 force field. When simulating with AMBER99SB and CHARMM27 the encounter complexes did not yield a comparable amount of β-sheet structure, instead the peptides sampled coil conformations. From multiple trajectories we found that the docked dimers with only few contacts frequently detached irreversibly after a finite time, especially if no interpeptide interactions involving backbone hydrogen bonds were present from the start. This can be seen from the different behavior and kinetic stability of enc1 and enc2 of the IB12 peptide in the different force fields. Partition properties of simple organic analogs of occurring amino acids show that GROMOS96 43A1 force field parameters induce less affinity to water for several polar amino acids (14). However, reasonable accuracy for the free energy of solvation for hydrophobic amino acids (Ala, Val, Ile, Leu) and aromatic amino acids (Tyr) is achieved, which are the components of the mainly hydrophobic peptides (VQIVYK, VEALYL) investigated in the present study. Nguyen et al. (15) reported the eventual incorporation of free amyloid beta peptide monomers onto preformed oligomeric aggregates after a rapid docking with a comparable setup (GROMOS96 force field, spc explicit solvent). The authors concluded that this dock-and-lock mechanism is consistent with an earlier proposed kinetic experiment (16). For the hydrophilic GNNQQNY peptide a reproducible oligomer formation and stable pairs of β-sheets were previously reported in MD simulation studies with the GROMOS96 43A1 force field (15, 17, 18).

The similar secondary structure preference for the tested force fields, as well as the similar observed stability of pre-formed dimers provides confidence in the applied simulation protocol. Nevertheless, remaining differences on the level of dimeric encounter complexes stress the importance of continued force field development and validation.

References

1. Lindahl E, Hess B, van der Spoel D (2001) Gromacs 3.0: A package for molecular simulation and trajectory analysis. J Mol Mod 7: 306-317.

2. van der Spoel D, Lindahl E, Hess B, Groenhof G, Mark AE, et al. (2005) Gromacs: Fast, flexible, and free. J Comput Chem 26: 1701–1718.

3. Kutzner C, van der Spoel D, Fechner M, Lindahl E, Schmitt UW, et al. (2007) Speeding up parallel gromacs on high-latency networks. J Comput Chem 28: 2075-2084.

4. Hess B, Kutzner C, Van Der Spoel D, Lindahl E (2008) Gromacs 4.0: algorithms for highly efficient, load-balanced, and scalable molecular simulation. J Chem Theory Comput 4: 435–447.

5. Mackerell AD, Feig M, III CLB (2004) Extending the treatment of backbone energetics in protein force fields: Limitations of gas-phase quantum mechanics in reproducing protein conformational distributions in molecular dynamics simulations. J Comput Chem 25: 1400-1415.

6. Hornak V, Abel R, Okur A, Strockbine B, Roitberg A, et al. (2006) Comparison of multiple amber force fields and development of improved protein backbone parameters. Proteins 65: 712–725.

7. Bjelkmar P, Larsson P, Cuendet MA, Hess B, Lindahl E (2010) Implementation of the charmm force field in gromacs: Analysis of protein stability rffects from correction maps, virtual interaction sites, and water models. J Chem Theory Comput 6: 459–466.

8. Sorin EJ, Pande VS (2005) Exploring the helix-coil transition via all-atom equilibrium ensemble simulations. Biophys J 88: 2472–2493.

9. Best RB, Hummer G (2009) Optimized molecular dynamics force fields applied to the helix-coil transition of polypeptides. J Phys Chem B 113: 9004-9015.

10. Joung IS, Cheatham TE (2008) Determination of alkali and halide monovalent ion parameters for use in explicitly solvated biomolecular simulations. J Phys Chem B 112: 9020-9041.

11. Darden T, York D, Pedersen L (1993) Particle mesh ewald: An n-log(n) method for ewald sums in large systems. J Chem Phys 98: 10089–10092.

12. Essmann U, Perera L, Berkowitz ML, Darden T, Lee H, et al. (1995) A smooth particle mesh ewald method. J Chem Phys 103: 8577-8593.

13. Matthes D, de Groot B (2009) Secondary structure propensities in peptide folding simulations: A systematic comparison of molecular mechanics interaction schemes. Biophys J 97: 599-608.

14. Villa A, Mark AE (2002) Calculation of the free energy of solvation for neutral analogs of amino acid side chains. J Comput Chem 23: 548–553.

15. Nguyen PH, Li MS, Stock G, Straub JE, Thirumalai D (2007) Monomer adds to preformed structured oligomers of abeta peptides by a two-stage dock-lock mechanism. Proc Natl Acad Sci U S A 104: 111-116.

16. Esler WP, Stimson ER, Jennings JM, Vinters HV, Ghilardi JR, et al. (2000) Alzheimer's disease amyloid propagation by a template-dependent dock-lock mechanism? Biochemistry 39: 6288-6295.

17. Zhang Z, Chen H, Bai H, Lai L (2007) Molecular dynamics simulations on the oligomer-formation process of the gnnqqny peptide from yeast prion protein sup35. Biophys J 93: 1484–1492.

18. Esposito L, Pedone C, Vitagliano L (2006) Molecular dynamics analyses of cross-beta-spine steric zipper models: beta-sheet twisting and aggregation. Proc Natl Acad Sci U S A 103: 11533–11538.
